# γ-TuRCs and the augmin complex are required for the development of highly branched dendritic arbors in *Drosophila*

**DOI:** 10.1242/jcs.261534

**Published:** 2024-05-10

**Authors:** Amrita Mukherjee, Yaiza Andrés Jeske, Isabelle Becam, Anaelle Taïeb, Paul Brooks, Joanna Aouad, Clementine Monguillon, Paul T. Conduit

**Affiliations:** ^1^ Department of Zoology, University of Cambridge, Downing Street, Cambridge CB2 3EJ, UK; ^2^ MRC Toxicology Unit, Gleeson Building, Tennis Court Road, Cambridge CB2 1QR, UK; ^3^ Université Paris Cité, CNRS, Institut Jacques Monod, F-75013 Paris, France

**Keywords:** Augmin, *Drosophila*, γ-TuRC, Microtubules, Neurons

## Abstract

Microtubules are nucleated by γ-tubulin ring complexes (γ-TuRCs) and are essential for neuronal development. Nevertheless, γ-TuRC depletion has been reported to perturb only higher-order branching in elaborated *Drosophila* larval class IV dendritic arborization (da) neurons. This relatively mild phenotype has been attributed to defects in microtubule nucleation from Golgi outposts, yet most Golgi outposts lack associated γ-TuRCs. By analyzing dendritic arbor regrowth in pupae, we show that γ-TuRCs are also required for the growth and branching of primary and secondary dendrites, as well as for higher-order branching. Moreover, we identify the augmin complex (hereafter augmin), which recruits γ-TuRCs to the sides of pre-existing microtubules, as being required predominantly for higher-order branching. Augmin strongly promotes the anterograde growth of microtubules in terminal dendrites and thus terminal dendrite stability. Consistent with a specific role in higher-order branching, we find that augmin is expressed less strongly and is largely dispensable in larval class I da neurons, which exhibit few higher-order dendrites. Thus, γ-TuRCs are essential for various aspects of complex dendritic arbor development, and they appear to function in higher-order branching via the augmin pathway, which promotes the elaboration of dendritic arbors to help define neuronal morphology.

## Teaser

**Highlighted Article:** Analyzing neurons at different developmental stages reveals a role for γ-TuRCs in many aspects of dendrite arbor development and a more specific role for augmin in high-order dendritic branching.

## INTRODUCTION

Microtubules are essential cytoskeletal components necessary for neuronal growth and function (Kapitein and Hoogenraad, 2015; Rolls et al., 2021). These polarized filaments run throughout axons and dendrites performing at least two important functions. They provide tracks for molecular motors to traffic cargo between the soma and neurite terminals, and their outward growth supports and facilitates neurite growth and branching. Within axons, most microtubules have their plus ends pointing away from the soma, referred to as plus-end-out microtubules, whereas dendrites contain either mixed or predominantly minus-end-out microtubules (Rolls and Jegla, 2015). This difference in microtubule polarity is important for the correct polarized trafficking of cargoes into axons and dendrites and thus for axon and dendrite identity (Rolls, 2011; Tas et al., 2017; Thyagarajan et al., 2022).

The *de novo* formation of microtubules is kinetically unfavorable at physiological concentrations of tubulin dimers. Thus, microtubule nucleation is promoted by microtubule-associated proteins (MAPs) or protein complexes that locally concentrate tubulin dimers (Tovey and Conduit, 2018). The best-characterized nucleator is the multi-protein γ-tubulin ring complex (γ-TuRC), which templates the lateral assembly of tubulin dimers to form a 13-protofilament microtubule that extends with its plus-end growing away from the γ-TuRC (Kollman et al., 2011; Brito et al., 2024). γ-TuRC activity is normally low in the cytosol but increases on recruitment to specific sites called microtubule-organizing centers (MTOCs) (Sanchez and Feldman, 2016; Tillery et al., 2018). Centrosomes are the best-known and best-characterized MTOC and are very active during mitosis (Conduit et al., 2015), but can be inactivated in neurons, particularly after early developmental stages (Nguyen et al., 2011; Stiess et al., 2010). Thus, non-centrosomal MTOCs play an important role in organizing the neuronal microtubule cytoskeleton (Lüders, 2020). So far, the surface of Golgi structures (Fu et al., 2019; Ori-McKenney et al., 2012; Valenzuela et al., 2020; Yalgin et al., 2015; Yang and Wildonger, 2020; Zhou et al., 2014), endosomes (Weiner et al., 2020) and centrosomal material in the distal tip of ciliated neurons (Garbrecht et al., 2021; Harterink et al., 2018; Magescas et al., 2021) have been reported as non-centrosomal MTOCs in *Drosophila melanogaster* and *Caenorhabditis elegans* neurons. Microtubules are also nucleated from *en passant* boutons in cultured mammalian hippocampal neurons (Qu et al., 2019).

Another important mode of non-centrosomal microtubule nucleation that has been implicated in neuronal development is nucleation via the augmin complex (hereafter augmin; known as the HAUS complex in mammals). Each individual augmin complex recruits an individual γ-TuRC to the side of a pre-existing microtubule to nucleate an individual microtubule with the same polarity as the mother microtubule (Goshima et al., 2008; Kamasaki et al., 2013; Lawo et al., 2009; Liu et al., 2014; Petry et al., 2013; Song et al., 2018; Verma and Maresca, 2019). These sparsely distributed nucleation events can be parallel or branched depending on the cell type and the presence or absence of certain protein and protein modifications (Sánchez-Huertas and Lüders, 2015). Augmin subunits were first identified in a screen for factors required for spindle assembly in *Drosophila* S2 cells and were shown to be important for microtubule generation within the mitotic spindle (Goshima et al., 2007, 2008). This was consistent with previous observations of microtubule nucleation from pre-existing microtubules within the cortical network of plant and fission yeast interphase cells (Janson et al., 2005; Murata et al., 2005; Wasteneys and Williamson, 1989), which was subsequently also observed in *Xenopus* egg extracts (Petry et al., 2013). The molecular architecture of augmin has now been revealed by a combination of cryo-electron microscopy (EM) and computational predictions, providing molecular details on how these complexes simultaneously bind microtubules and γ-TuRCs (Gabel et al., 2022; Zupa et al., 2022). Direct observations of single augmin-mediated nucleation events have also been made using total internal reflection fluorescence (TIRF) microscopy in *Drosophila* S2 cells (Verma and Maresca, 2019). In mammalian neurons, augmin depletion affects cortical neuron migration and polarization in mice, and microtubule density and polarity in the dendrites and axons, respectively, of cultured rat hippocampal neurons (Cunha-Ferreira et al., 2018; Sánchez-Huertas et al., 2016). Nevertheless, whether neurons have a general requirement for augmin, or whether it is required in specific settings or for specific processes remains unclear.

*Drosophila* larval dendritic arborization neurons (da neurons) are a well-established model system to study neuronal morphology and microtubule regulation *in vivo* (Jan and Jan, 2010). They form in embryos but continue to develop throughout the larval stages until pupation. They exhibit elaborate but stereotypical dendritic arbors that grow mainly in two dimensions in the larval body wall, making them amenable to morphology analysis and accessible to live imaging *in situ*. They are genetically tractable with well-characterized neuron-type-specific Gal4 drivers able to express UAS-controlled fluorescent reporters and RNAi constructs. Da neurons are split into four classes (Grueber et al., 2002), with simple class I and elaborate class IV neurons most commonly studied. Class I neurons are proprioceptive and comprise a relatively long primary dendrite that branches repeatedly into multiple secondary dendrites that extend along the antero-posterior axis to sense stretch during larval crawling (Song et al., 2007; Vaadia et al., 2019). These secondary dendrites generate few higher-order dendritic branches. In contrast, class IV da neurons are nociceptive (e.g. Hu et al., 2017; Xiang et al., 2010; Zhong et al., 2010) and tile the body wall by branching extensively (Grueber et al., 2002). They comprise long primary and secondary dendrites that branch into many tertiary and terminal (higher-order) branches to cover their receptive field.

Within the dendrites of class I and class IV da neurons, it has been proposed that γ-TuRCs associate with fragments of Golgi called Golgi outposts to mediate de-centralized microtubule nucleation events that regulate microtubule polarity and arbor morphology (Ori-McKenney et al., 2012; Yalgin et al., 2015). The main phenotype after γ-tubulin depletion from class IV neurons was a reduction in the number and length of higher-order branches and it was concluded that this was due to defects in γ-TuRC-mediated microtubule nucleation from Golgi outposts (Ori-McKenney et al., 2012). However, the conclusion that Golgi outposts help regulate microtubule polarity in da neurons was later challenged by findings showing that exogenously expressed γ-tubulin–GFP localizes to branch points independently of Golgi outposts and that microtubule polarity was unaffected by the re-positioning of Golgi outposts out of dendrites (Nguyen et al., 2014). Using endogenously regulated reporters of γ-TuRCs, it was then shown that γ-TuRCs localize to relatively few Golgi outposts that are present only within primary branch points (close to the soma) (Mukherjee et al., 2020). These γ-TuRC-positive Golgi outposts are not directly associated with terminal branches, suggesting that the loss of terminal branches after γ-tubulin depletion might represent a failure in an as-yet-unidentified, Golgi outpost-independent, mode of microtubule nucleation. In addition, it has been reported that the long primary and secondary dendrites that make up the main skeleton of the class IV arbors develop rather normally after γ-tubulin depletion (Ori-McKenney et al., 2012). It remains unclear, however, whether γ-TuRCs were efficiently depleted from the neurons in these experiments.

In this study, we have taken advantage of class IV arbor regrowth during pupal development where we find that the effects of RNAi are stronger than in larvae. By 18 h after pupal formation (APF) class IV da neuron dendritic arbors are completely removed in a process called pruning, leaving the neuronal cell body and axon intact (Kuo et al., 2005; Williams and Truman, 2005). The class IV da neuron dendritic arbors then have to regrow during pupal development to re-fill their receptive fields in preparation for adulthood (Kuo et al., 2005; Satoh et al., 2012; Shimono et al., 2009). Although microtubule regulation in da neurons is normally analyzed in larvae, the regrowth phase in pupae provides an opportunity to monitor the entirety of dendritic arbor growth when RNAi constructs have been expressed for several days and when there is less chance of persistent maternal proteins negating phenotypes.

Indeed, by analyzing re-growing pupal class IV dendritic arbors expressing RNAi constructs against γ-TuRC components we show that γ-TuRCs are essential for many aspects of class IV arbor development, including the growth and branching of primary and secondary dendrites and for higher-order branching. These phenotypes are much less severe in larvae, but can be detected when using the most efficient RNAi line against γ-TuRCs. We also show that depletion of augmin predominantly affects higher-order branching in both pupal and larval class IV neurons. In larvae, we observe defects in microtubule polarity in distal secondary and tertiary dendrites and a reduction in the frequency of anterograde microtubule growth within terminal dendrites after augmin depletion. This correlates with a reduction in the stability of these terminal dendrites, which is thought to be microtubule dependent, but not in their initial growth, which is actin dependent. Consistent with a specialized role for augmin in higher-order branching, it is expressed more strongly in larval class IV neurons than in class I neurons, which have very few higher-order dendrites, and its depletion from class I neurons has little effect on arbor morphology. Given the known role and mode of operation of augmin in other cell types, our findings suggest that augmin mediates sparsely distributed microtubule nucleation events within the dendritic arbor of class IV da neurons that provide sufficient numbers of microtubules to stabilize the nascent growth of higher-order dendrites. Our data also suggest that class IV and class I da neurons use different modes of microtubule nucleation to support the differential growth of their dendritic arbors.

## RESULTS

### Depletion of γ-TuRCs strongly perturbs the development of pupal class IV da neurons

**Fig. 1.**
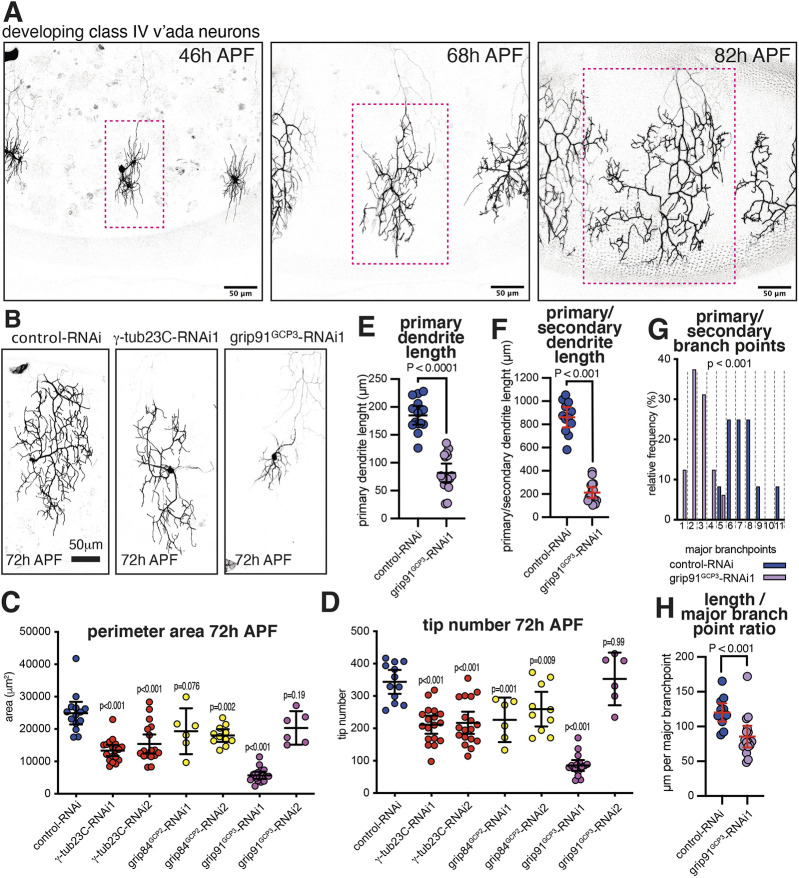
**γ-TuRC depletion results in severe dendritic morphology defects in the dendritic arbors of pupal class IV neurons.** (A) Confocal images show pupal class IV v'ada neurons expressing CD4–tdGFP at different times APF, as indicated, using ppk-Gal4. The red dotted box indicates a single dendritic field. Note that the neurons are from different animals but were imaged within the same abdominal segment. (B) Confocal images show pupal class IV v'ada neurons at 72 h APF expressing CD4–tdGFP, UAS-Dicer2 and different UAS-RNAi lines, as indicated, using ppk-Gal4. (C,D) Graphs showing the perimeter area of dendritic arbors (μm^2^) (C) and the number of dendrite tips (D) of pupal class IV v'ada neurons at 72 h APF expressing CD4–tdGFP, UAS-Dicer2 and different UAS-RNAi lines, as indicated, using ppk-Gal4. Each point on the graph represents a different neuron. *n*=14, 18, 18, 6, 11, 17, 6 for the datasets from left to right on the graph in C, and *n*=12, 18, 18, 6, 10, 17, 6 for the datasets from left to right on the graph in D. The mean±95% c.i. is indicated. *P*-values are indicated (one-way ANOVA with Dunnett's multiple comparisons test was used to compare the control dataset to each experimental dataset). Note how most γ-TuRC RNAi constructs lead to a reduction in arbor area and tip number but that expressing grip91^GCP3^-RNAi1 results in the strongest reduction in both indicators. (E,F) Graphs showing the lengths of the longest primary dendrites (E) or the combined length of primary and secondary dendrites (F) for control-RNAi (*n*=14) and grip91^GCP3^-RNAi1 (*n*=17) neurons. The mean±95% c.i. is indicated. Two-sided unpaired *t*-tests were used to compare the datasets. (G) Graph showing the relative proportion of primary and secondary branch points for control-RNAi (*n*=13) and grip91^GCP3^-RNAi1 (*n*=16) neurons. Each segment represents the percentage for a given number of branch points. A two-sample Kolmogorov–Smirnov test was used to compare the datasets. (H) Graph showing the ratio between primary and secondary dendrite length and branch point number for control-RNAi (*n*=13) and grip91^GCP3^-RNAi1 (*n*=16) neurons. The mean±95% c.i. is indicated. A two-sided unpaired *t*-test was used to compare the datasets.

We began by establishing imaging conditions for class IV da neurons during pupal development. We chose to analyze v'ada class IV neurons as their dendritic arbor development has been previously described (Yoong et al., 2020) and they remain free from overlap by other fluorescent cells. Dendrites initially form at ∼28–30 h APF and then undergo outgrowth and branching up until ∼80 h APF (Fig. 1A). Similar to what is seen with larval ddaC neurons, primary and secondary dendrites make up the backbone of the arbor in pupae and higher-order branches help fill the dendritic field (Fig. S1). We tested the effect of depleting γ-TuRCs on the growth and development of these arbors by expressing various UAS-controlled RNAi constructs in the presence of UAS-Dicer2 using the class IV-specific driver ppk-Gal4. We chose to perform arbor morphology analysis at ∼72 h APF because at this stage the arbors had grown to a convenient size for analysis. Of all the γ-TuRC components, only γ-tubulin, Grip84 and Grip91, members of the γ-tubulin small complex (γ-TuSC), are essential for viability and conserved throughout eukaryotes (Tovey and Conduit, 2018). We therefore tested two independent RNAi lines for each of these essential γ-TuRC components (Table S1). There are two γ-tubulin genes in *Drosophila*, a maternally expressed form called γ-tubulin37C and a zygotically expressed form called γ-tubulin23C. RNAi targeting γ-tubulin37C can therefore be used as a negative control within da neurons (Feng et al., 2019; Mukherjee et al., 2020) and was used throughout this study. For clarity, we have added the names of the Grip84 and Grip91 mammalian homologs in superscript (i.e. Grip84^GCP2^ and Grip91^GCP3^; GCP2 and GCP3 are also known as TUBGCP2 and TUBGCP3).

For each condition, we measured both the number of dendrite tips and the arbor area (defined by drawing a line around the perimeter of the dendritic arbor – hereafter perimeter area – see Fig. S1A and the Materials and Methods section) at 72 h APF. All but one RNAi construct resulted in a significant reduction in tip number compared to the control, and all but two resulted in a significant reduction in perimeter area (Fig. 1B–D). As expected, phenotypic strength varied, presumably due to differences in RNAi efficiency, with the strongest reduction in both perimeter area and tip number resulting from expressing grip91^GCP3^-RNAi1 [corresponding to Vienna *Drosophila* Resource Center (VDRC) KK library construct 104667] and the second strongest resulting from expressing γ-tubulin23C-RNAi1, which was previously used when assessing phenotypes in larvae (Ori-McKenney et al., 2012) (Fig. 1B-D). Although we cannot rule out that the stronger phenotype observed with grip91^GCP3^-RNAi1 is due to unintended depletion of another gene, grip91^GCP3^-RNAi1 has no predicted off-targets (VDRC website). Moreover, we confirmed by RT-qPCR on wing disc samples that grip91^GCP3^-RNAi1 was able to deplete *grip91* mRNA (Fig. S2) and that expression in class I da neurons strongly reduced the presence of endogenously tagged γ-tubulin–GFP at Golgi mini-stacks within the soma (Fig. S3). Thus, expressing grip91^GCP3^-RNAi1 has a strong effect on γ-TuRCs in da neurons.

We therefore further analyzed pupal class IV neurons expressing grip91^GCP3^-RNAi. Strikingly, compared to controls, there was a ∼75% and ∼77% reduction in dendritic tips and perimeter area at 72 h APF, respectively, when expressing grip91^GCP3^-RNAi (Fig. 1B–D). The length of the longest primary dendrite that extends from the soma was reduced by ∼55% (Fig. 1E) and the combined length of primary and secondary dendrites was reduced by ∼73% (Fig. 1F). There was also a strong reduction in the combined total number of primary and secondary branchpoints (Fig. 1G). Nevertheless, these major branchpoints occurred more frequently per dendrite length in grip91^GCP3^-RNAi neurons (Fig. 1H), suggesting that although the primary and secondary dendrites of grip91^GCP3^-RNAi neurons struggle to both grow (Fig. 1F) and branch (Fig. 1G), they perhaps struggle more to grow than to branch. Taking the data together, we conclude that γ-TuRCs are required for primary and secondary dendrite growth and branching, and for higher-order branching.

### Depletion of γ-TuRCs perturbs the development of larval class IV da neurons, albeit more mildly than in pupal neurons

We considered that the phenotypes we observed in pupal neurons were stronger than those previously observed in larval neurons because of a maternal contribution of γ-TuRCs (provided by the mother to the embryo) that persists during larval stages. This possibility had already been suggested by others (Ori-McKenney et al., 2012) and is supported by our unpublished observations that *γ-tubulin23c*-null mutant flies can survive until late larval/early pupal stages. RNAi expression might also be relatively low during the early stages of arbor development in larvae, and the previously observed phenotypes in larvae might have been stronger if the most efficient γ-TuRC RNAi line had been used.

**Fig. 2.**
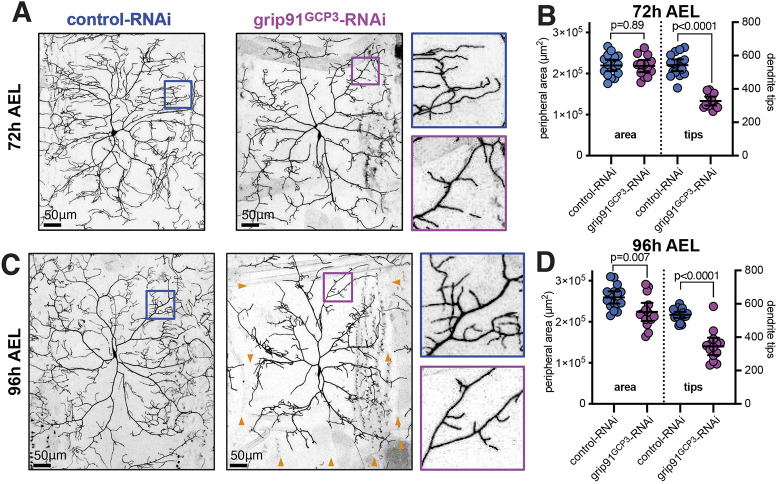
**γ-TuRC depletion affects higher-order branching at 72 h AEL but both primary and secondary dendrite growth and higher-order branching at 96 h AEL.** (A,C) Confocal images show larval class IV ddaC neurons at either 72 h AEL (A) or 96 h AEL (C) expressing CD4–tdGFP, UAS-Dicer2 and either control-RNAi or grip91^GCP3^-RNAi, as indicated, using ppk-Gal4. Enlargements of areas for control-RNAi (blue) and grip91^GCP3^-RNAi (magenta) are shown on the right of each panel. Orange arrowheads in C indicate large gaps between adjacent neurons. Scale bars are indicated within the images. (B,D) Graphs showing the perimeter area of dendritic arbors (μm^2^) (left axes) and the number of dendrite tips (right axes) of larval class IV ddaC neurons at either 72 h AEL (B) or 96 h AEL (D) expressing CD4–tdGFP, UAS-Dicer2 and either control-RNAi or grip91^GCP3^-RNAi, as indicated, using ppk-Gal4. Each point on the graph represents a different neuron, *n*=16 and 13 for control-RNAi and grip91^GCP3^-RNAi, respectively, in B, *n*=16, 14, 18, and 14 for the datasets from left to right in the graph in D. The mean±95% c.i. is indicated. Two-sided unpaired *t*-tests were used to compare the datasets. *P*-values are indicated for each comparison.

To examine the effect of depleting γ-TuRCs on larval class IV neurons, we expressed grip91^GCP3^-RNAi with UAS-Dicer2 and examined class IV ddaC arbors at 72 h and 96 h after egg laying (AEL). The growth of class IV da neurons has been well documented (Gao et al., 1999; Parrish et al., 2009; Shree et al., 2022). Dendrite growth begins in late embryos at around 16 h AEL. During the first and second larval instar stages, from 24 h to 72 h AEL, the dendritic arbors grow faster than the hemisegment boundaries until they fill the hemisegments at ∼72 h AEL. The arbors then grow at the same rate as the hemisegments, termed scaling growth, until 96 h AEL. At 96–120 h AEL, the wandering third-instar stage, the arbors occupy the same area but continue to add internal branches, maximizing their density and coverage of the dendritic field. We found that there was no significant difference in perimeter area between control and grip91^GCP3^-RNAi arbors at 72 h AEL (Fig. 2A,B), suggesting that the growth of the main primary and secondary dendrites is unaffected. There was, however, a ∼40% reduction in dendrite tips (Fig. 2A,B), showing that higher-order branches failed to properly form or be maintained. At 96 h AEL, whereas the control arbors had scaled with segment growth and increased their perimeter area by ∼15%, grip91^GCP3^-RNAi arbors failed to scale and remained at a similar perimeter area to the arbors at 72 h AEL, while also having a ∼35% reduction in dendrite tips (Fig. 2C,D). The failure to scale was made obvious by the appearance of large gaps between adjacent grip91^GCP3^-RNAi neurons (orange arrowheads, Fig. 2C). Thus, expressing grip91^GCP3^-RNAi perturbs the growth and/or stability of higher-order dendrites at both 72 h and 96 h AEL, but takes effect on the outward growth of primary and secondary dendrites only after 72 h AEL, during scaling growth. These data are consistent with an inefficiency of RNAi during early dendritic arbor growth, particularly concerning primary and secondary dendrite growth.

Collectively, our data from pupal and larval neurons show that γ-TuRCs are required for the proper growth and development of class IV dendritic arbors, with their depletion affecting primary and secondary dendrite growth (during later larval stages and throughout pupal development) and the growth and/or stability of the more dynamic higher-order branches.

### Depletion of augmin perturbs dendritic branching of pupal class IV da neurons

Collating the finding above that γ-TuRC depletion perturbs higher-order branching in class IV neurons with the previous observation that distal Golgi outposts lack γ-TuRCs (Mukherjee et al., 2020), we considered which other modes of γ-TuRC-mediated microtubule nucleation might be involved in regulating higher-order branching. The augmin complex was a good candidate, as it recruits γ-TuRCs to the sides of pre-existing microtubules from which new daughter microtubules are nucleated. This mode of nucleation could in theory occur throughout the arbor. Moreover, these nucleation events can be angled up to ∼60˚ depending on the organism and cell type [∼36˚ in *Drosophila* S2 cells (Verma and Maresca, 2019)], which could provide a mechanism to promote microtubule growth into nascent higher-order dendrites.

**Fig. 3.**
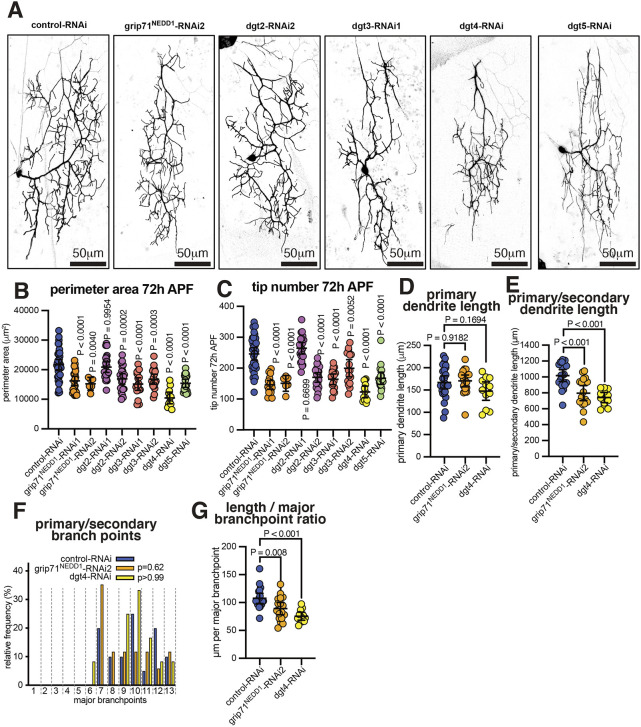
**Augmin depletion reduces higher-order branching in the dendritic arbors of pupal class IV neurons.** (A) Confocal images show pupal class IV v'ada neurons at 72 h APF expressing CD4–tdGFP, UAS-Dicer2 and different UAS-RNAi lines, as indicated, using ppk-Gal4. Scale bars are indicated within the images. (B,C) Graphs showing the perimeter area of dendritic arbors (μm^2^) (B) and the number of dendrite tips (C) of pupal class IV v'ada neurons at 72 h APF expressing CD4–tdGFP, UAS-Dicer2 and different UAS-RNAi lines, as indicated, using ppk-Gal4. Each point on the graph represents a different neuron. *n*=36, 24, 23, 23, 17, 19, 12, and 18 for the datasets from left to right in the graph in B, and *n*=33, 19, 6, 22, 22, 17, 16, 12, and 18 for the datasets from left to right in the graph in C. The mean±95% c.i. is indicated. *P*-values are indicated (one-way ANOVA with Dunnett's multiple comparisons test was used to compare the control dataset to each experimental dataset). Note how most augmin RNAi constructs lead to a reduction in arbor area and tip number but that expressing dgt4-RNAi results in the strongest reduction in both indicators. (D,E) Graphs showing the lengths of the longest primary dendrites (D) or the combined length of primary and secondary dendrites (E) for control-RNAi (*n*=20), grip71^NEDD1^-RNAi1 (*n*=17) and dgt4-RNAi (*n*=12) neurons. The mean±95% c.i. is indicated. One-way ANOVA with Dunnett's multiple comparisons test was used to compare the control dataset to each experimental dataset. (F) Graph showing the relative proportion of primary and secondary branch points for control-RNAi (*n*=20), grip7^NEDD1^-RNAi1 (*n*=17) and dgt4-RNAi (*n*=12) neurons. Each segment represents the percentage for a given number of branch points. A Kruskal–Wallis test was used to compare each condition to the control. (G) Graph showing the ratio between primary and secondary dendrite length and branch point number for control-RNAi (*n*=20), grip7^NEDD1^-RNAi1 (*n*=17) and dgt4-RNAi (*n*=12) neurons. The mean±95% c.i. is indicated. One-way ANOVA with Dunnett's multiple comparisons test was used to compare the datasets.

We first tested whether augmin components were expressed in class IV da neurons. We crossed a UAS-myr-GFP line (GFP targeted to membrane) to a publicly available enhancer trap line where Gal4 had been inserted into the gene region of the augmin component Dgt5 (BL77584). We observed GFP signal within 35/35 larval class IV ddaC neurons from six different animals (Fig. S4), consistent with the expression of the augmin complex. We then examined the effects on pupal neurons of expressing RNAi constructs targeting augmin. Augmin comprises eight different proteins and its ability to recruit γ-TuRCs to spindle microtubules depends on the γ-TuRC adapter protein Grip71 (the homolog of NEDD1). We therefore expressed different RNAi lines against various augmin components (*dgt2*, *dgt3*, *dgt4* or *dgt5*) or grip71^NEDD1^ (Table S2). We confirmed by PCR that none of the VDRC KK lines contained the aberrant 40D insertion (data not shown). All but one of the eight RNAi lines, including both grip71^NEDD1^-RNAi lines, caused a significant reduction in dendrite tip number and perimeter area (Fig. 3A–C), consistent with a role for augmin in mediating class IV dendritic arbor growth. The strongest effect came when expressing Dgt4-RNAi, which resulted in a ∼52% reduction in perimeter area (Fig. 3B) and a ∼50% reduction in tip number (Fig. 3C), and was capable of depleting *dgt4* mRNA when expressed in wing discs (Fig. S2). In contrast to the effects after γ-TuRC depletion, however, there was no significant reduction in the length of the longest primary dendrite (Fig. 3D), and there was only a ∼27% reduction in the total length of primary and secondary branches (compared to a ∼73% reduction in grip91^GCP3^-RNAi neurons) (Fig. 3E). Moreover, there was no effect on the number of primary and secondary branchpoints (Fig. 3F), meaning that the length of primary and secondary dendrite per branchpoint was reduced slightly (Fig. 3G). Thus, although there is some effect on primary and secondary dendrite growth, much of the reduction in peripheral arbor area and tip number after augmin depletion appears to be due to defects in higher-order branching. Depleting the γ-TuRC component Grip71^NEDD1^ produced very similar phenotypes (Fig. 3B–G), suggesting that Grip71^NEDD1^ functions specifically in the augmin pathway in these neurons, rather than acting as a general γ-TuRC targeting protein.

### Depletion of augmin perturbs dendritic branching within larval class IV da neurons

**Fig. 4.**
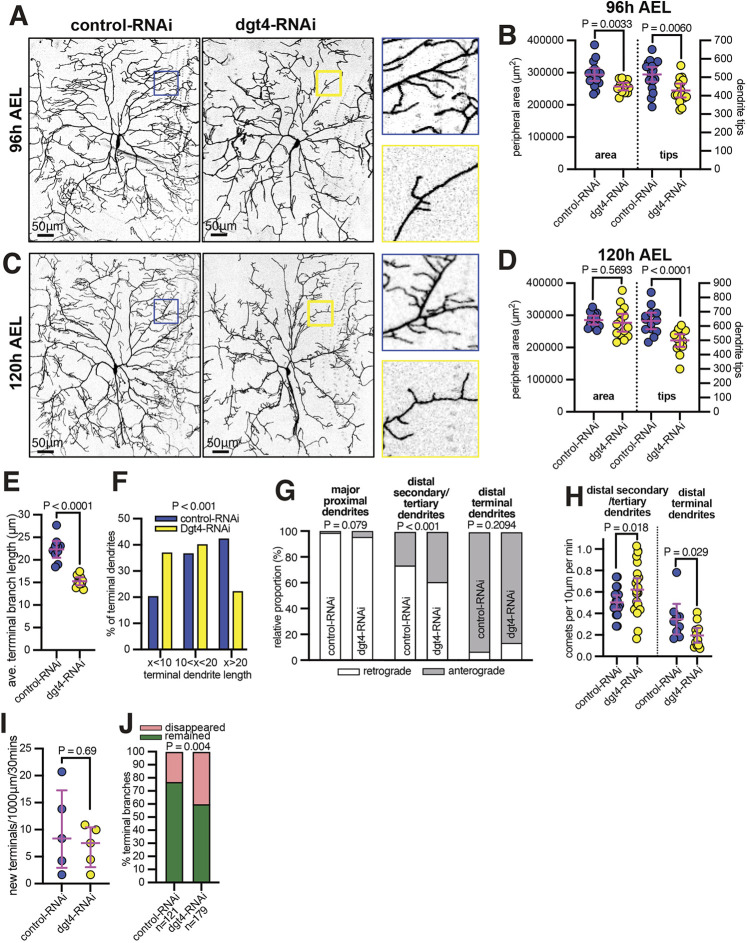
**Augmin depletion affects higher-order branching in larval class IV da neurons.** (A,C) Confocal images show larval class IV ddaC neurons at either 96 h AEL (A) or 120 h AEL (C) expressing CD4–tdGFP, UAS-Dicer2 and either control-RNAi or dgt4-RNAi, as indicated, using ppk-Gal4. Enlargements of areas for control-RNAi (blue) and dgt4-RNAi (yellow) are shown on the right of each panel. Scale bars are indicated within the images. (B,D) Graphs showing the perimeter area of dendritic arbors (μm^2^) (left axes) and the number of dendrite tips (right axes) of larval class IV ddaC neurons at either 96 h AEL (B) or 120 h AEL (D) expressing CD4–tdGFP, UAS-Dicer2 and either control-RNAi or dgt4-RNAi, as indicated, using ppk-Gal4. Each point on the graph represents a different neuron. *n*=17, 13, 18, 15 for the datasets from left to right on the graph B, and *n*=13, 14, 13, 15 for the datasets from left to right on the graph D. The mean±95% c.i. is indicated. Two-sided unpaired *t*-tests were used to compare the datasets. *P*-values are indicated for each comparison. (E) Graph showing the average lengths of terminal dendrites for control and dgt4-RNAi neurons. Each data point represents the average value from an individual neuron. *n*=10 neurons for both conditions. The mean±95% c.i. is are indicated. A two-sided unpaired *t*-test was used to compare the datasets. (F) Graph showing the relative proportion of terminal dendrites of a given length (as indicated below) from control-RNAi (blue) or dgt4-RNAi (purple) neurons. Number of dendrites analyzed: 726 for control-RNAi and 536 for dgt4-RNAi. Note the shift in distribution towards shorter dendrites in dgt4-RNAi neurons. (G) Graphs showing the proportion of retrograde (white) or anterograde (gray) EB1–GFP comets in major proximal dendrites, distal secondary dendrites, distal tertiary dendrites or distal terminal dendrites, as indicated. *n*=(dendrites;neurons): (216;6), (201;7), (101;5), (208;7), (89;7), (248;7), (45;7), (37;7) for the datasets from left to right on the graph. Fisher's exact tests were used to compare the proportions. *P*-values are indicated above the graphs. (H) Graph showing the number of EB1–GFP comets per 10 µm per min in control-RNAi or dgt4-RNAi distal secondary dendrites, tertiary dendrites or terminal dendrites. Secondary and tertiary dendrites were grouped together as they displayed similar EB1–GFP comet frequencies. Each point on the graph represents a different neuron. *n*=9, 11, 9, 12 for the datasets from left to right on the graph. The mean±95% c.i. is indicated. Unpaired *t*-tests were used to compare control-RNAi and dgt4-RNAi neurons for each dendrite type. *P*-values are indicated above the graph. (I) Graph showing the frequency at which new terminal branches formed within control and dgt4-RNAi neurons during a period of 30 min. Each data point represents an individual neuron. *n*=5 neurons for both conditions. Median and interquartile range are indicated. A two-tailed Mann–Whitney test was used to compare the datasets. *P*-value indicated above the graph. (J) Graph showing the proportion of terminal dendrites that remain (green) or disappear (red) during the 30 min in either control-RNAi or dgt4-RNAi neurons, as indicated. A Fisher's exact test was used to compare the proportions. *P*-value indicated above the graph.

We next tested the effect of augmin depletion within larval class IV neurons. Although RNAi may be less efficient than in pupae, the dynamics of microtubules are well characterized in larval neurons (Ori-McKenney et al., 2012; Sears and Broihier, 2016), allowing us to carefully examine the effect of augmin depletion on microtubule polarity and growth. Moreover, we could compare effects between class I and class IV neurons, which have very different dendritic arbor morphologies. We expressed Dgt4-RNAi and UAS-Dicer2 within class IV da neurons using the ppk-gal4 driver and assessed dendritic arbor morphology at 96 h AEL. Compared to controls, there was an ∼17% and an ∼13% reduction in dendrite tip number and perimeter area, respectively (Fig. 4A,B). Intriguingly, however, the peripheral area was similar to that in controls 24 h later, at 120 h AEL, just before pupation, despite there still being a ∼22% reduction in dendrite tips (Fig. 4C,D). We also measured a ∼32% reduction in average terminal branch length in Dgt4-RNAi neurons at 96 h AEL (Fig. 4E), with a downward shift in the distribution of terminal dendrite length (Fig. 4F). These data are consistent with augmin having a more important role in higher-order branching than in the growth of primary and secondary dendrites, and this was made apparent by the presence of large areas void of higher-order dendrites within Dgt4-RNAi neurons (compare magnified views in Fig. 4A and C).

### Depletion of augmin perturbs microtubule polarity and frequency within the distal regions of larval class IV da neurons

To determine whether the reduction in higher-order branching of class IV da neurons correlated with defects in microtubule dynamics, we expressed the plus-end-tracking protein EB1–GFP in either control-RNAi or Dgt4-RNAi neurons (see Movies 1 and 2). EB1–GFP marks the growing ends of microtubules and is commonly used to determine microtubule polarity and new microtubule growth events. In class IV da neurons, the direction of EB1–GFP comet growth correlates with dendrite length, with comets becoming progressively more anterograde (growing away from the soma) with reduced branch length, which roughly correlates with dendrite order (primary, secondary, tertiary and terminal) (Ori-McKenney et al., 2012). Indeed, when imaging the long primary dendrites close to the soma we found that 213/216 (98.6%) of EB1–GFP comets were retrograde in control-RNAi neurons. Consistent with augmin not playing a role in the growth of primary dendrites, there was no significant decrease in the proportion of retrograde comets in these primary dendrites (192/201, 95.5%; *P*=0.079) (Fig. 4G).

When filming the more distal regions of control arbors, however, we could identify differences in comet polarity and frequency. In secondary and tertiary dendrites (see Fig. S1B for how dendrite order was established), there was a significant reduction in the proportion of retrograde comets in Dgt4-RNAi neurons: 73.8% (220/298) and 60.9% (380/622) of comets were retrograde in control-RNAi or Dgt4-RNAi neurons, respectively (*P*<0.001) (Fig. 4G). Thus, augmin depletion can affect microtubule polarity in *Drosophila* dendrites, as it does in mammalian axons (Cunha-Ferreira et al., 2018; Sánchez-Huertas et al., 2016). In contrast to secondary and tertiary dendrites, however, a higher proportion of retrograde comets was observed in the terminal dendrites of Dgt4-RNAi neurons compared to control neurons, although this was not statistically significant (Fig. 4G). When quantifying EB1–GFP comet frequency, we found, somewhat surprisingly, that distal secondary and tertiary dendrites displayed an ∼24% increase in EB1–GFP comets (Fig. 4H). In stark contrast, however, there was a ∼44% reduction in EB1–GFP comet frequency in distal terminal dendrites (Fig. 4H). The increase in comet frequency in distal secondary and tertiary dendrites could be due to the induction of neuronal stress, as can be the case after the depletion of other microtubule regulators (Feng et al., 2019). Alternatively, the reduction in microtubule growth in terminal dendrites might increase the availability of tubulin dimers for microtubule growth in distal secondary and tertiary dendrites. In either case, we conclude that augmin is required to maintain proper microtubule polarity in distal secondary and tertiary dendrites, and to promote anterograde microtubule growth within terminal dendrites.

### Terminal dendrites are destabilized in Dgt4-RNAi neurons

We next examined the dynamics of terminal dendrites in control-RNAi and Dgt4-RNAi neurons. It is known that terminal dendrites are highly dynamic (Shree et al., 2022), forming in an actin-dependent manner (Nithianandam and Chien, 2018; Stürner et al., 2019) and then either disappearing, remaining stable or extending their growth. Moreover, their stability correlates with the presence of anterograde EB1–GFP comets (Ori-McKenney et al., 2012; Sears and Broihier, 2016). We filmed distal regions of control-RNAi and Dgt4-RNAi neurons expressing a fluorescent membrane marker for 30 mins and found no significant difference in the rate at which new terminal dendrites formed (Fig. 4I; Movies 3, 4), consistent with augmin not being involved in actin remodeling. Strikingly, however, we found that the percentage of terminal dendrites that were present at the start of the movie but that disappeared by the end of the movie increased from 23% in control-RNAi neurons to 40% in Dgt4-RNAi neurons (Fig. 4J). Thus, the ability of augmin to promote anterograde microtubule growth within terminal dendrites appears to help stabilize these terminal dendrites, which presumably promotes higher-order branching.

### Augmin is largely dispensable for the development of larval class I da neurons

**Fig. 5.**
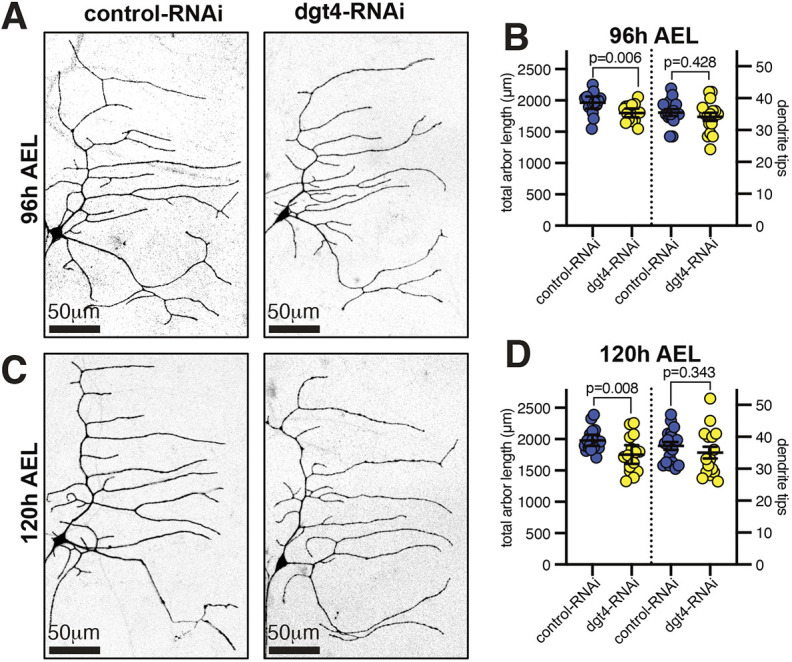
**Augmin depletion does not affect dendritic branching in larval class I da neurons.** (A,C) Confocal images show larval class I ddaE neurons at either 96 h AEL (A) or 120 h AEL (C) expressing CD4–tdGFP, UAS-Dicer2 and either control-RNAi or dgt4-RNAi, as indicated, using 221-Gal4. Scale bars are indicated within the images. (B,D) Graphs showing the total arbor length (μm) (left axes) and the number of dendrite tips (right axes) of larval class I ddaE neurons at either 96 h AEL (B) or 120 h AEL (D) expressing CD4–tdGFP, UAS-Dicer2 and either control-RNAi or dgt4-RNAi, as indicated, using 221-Gal4. Each point on the graph represents a different neuron. *n*=15, 16, 15, and 16 for the datasets from left to right on the graph B, and *n*=18, 16, 18, and 16 for the datasets from left to right on the graph D. The mean±95% c.i. is indicated. Two-sided unpaired *t*-tests were used to compare the datasets. *P*-values are indicated for each comparison.

Given that larval class I da neurons have only few higher-order dendrites and mainly comprise primary and secondary dendrites, we hypothesized that augmin might be largely dispensable for class I arbor development. Consistent with this, the dgt5 Gal4 enhancer trap was expressed less strongly in class I ddaE neurons than in class IV ddaC neurons (Fig. S4C). Moreover, expressing Dgt4-RNAi in class I neurons had little effect on dendritic arbor morphology. In Dgt4-RNAi neurons, there was only an ∼8.4% and ∼11.4% reduction in total arbor length at 96 h AEL (Fig. 5A,B) and 120 h AEL (Fig. 5C,D), respectively, showing that augmin is largely dispensable for the proper growth of class I da neuron primary and secondary dendrites (similar to what we found for class IV da neurons). There were also no significant changes in dendrite tip number at either 96 h (Fig. 5A,B) or 120 h (Fig. 5C,D) AEL. This is consistent with a previous observation that embryonic class I ddaE neurons within *wac* mutant flies (*wac* encodes an augmin subunit) also show no difference in dendrite tip number compared to controls (Yalgin et al., 2015). In control-RNAi and Dgt4-RNAi larval class I neurons, we measured an average of ∼36 dendrite tips at 120 h AEL, while Yalgin et al. measured an average of ∼20 dendrite tips during stage 17 of embryogenesis (just before hatching into larvae), ∼96 h earlier. Thus, class I ddaE neurons add, on average, ∼16 new dendrite branches during ∼96 h of larval development and this does not require augmin.

## DISCUSSION

Our data show that γ-TuRCs and augmin are required for the growth and development of highly branched class IV dendritic arbors. γ-TuRCs are required for all aspects of dendritic arbor development, whereas augmin appears to be required predominantly for higher-order branching. Consistent with this, augmin depletion has little effect on class I neurons, which have only few higher-order dendrites. Thus, although γ-TuRCs are generally required for dendritic arbor development, the augmin complex might be a key determinant of dendritic arbor morphology, promoting the elaboration of dendritic arbors.

Although we cannot exclude the possibility that augmin functions independently of γ-TuRCs in da neurons, a γ-TuRC-independent function of augmin has not been reported to our knowledge. Given the effects we observe on microtubule polarity and frequency after augmin depletion, our data are fully consistent with augmin functioning to recruit γ-TuRCs to the sides of microtubules to mediate microtubule nucleation events, as occurs in cycling cells (Song et al., 2018). We propose that these nucleation events occur throughout the arbor and lead to the anterograde growth of microtubules within terminal dendrites, which in turn help to stabilize the nascent growth of these dendrites.

**Fig. 6.**
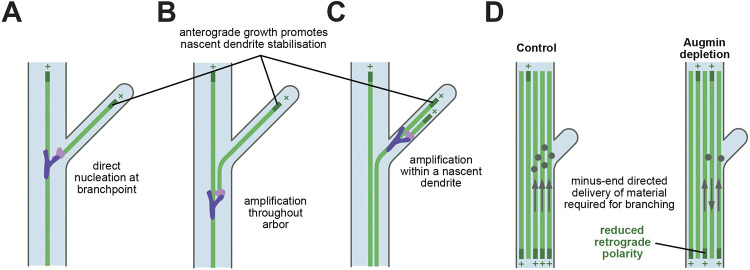
**Non-mutually exclusive possibilities for how augmin might promote higher-order dendritic branching.** Cartoons representing how augmin-mediated microtubule nucleation might promote the formation of terminal dendrites from either secondary or tertiary dendrites in the distal region of the class IV da neuron. (A) Augmin–γ-TuRC (purple and pink respectively) might assemble at the base of nascent dendrites and nucleate microtubules at angles that allow the daughter microtubules to grow into and stabilize the nascent dendrite. (B) Augmin–γ-TuRC might be required to amplify microtubules throughout the dendritic arbor, increasing the likelihood that microtubules turn into and stabilize nascent dendrites. (C) Augmin–γ-TuRC might be required to amplify anterograde microtubules within nascent dendrites, thereby promoting nascent dendrite stability. (D) Augmin might be required to maintain the correct polarity of microtubules within dendrites, thereby ensuring the correct motor-driven delivery along microtubules of material required for higher-order dendritic branching.

Our finding that augmin is required for higher-order branching in class IV neurons helps make sense of prior literature. Higher-order branching in larvae has been shown to be reduced after γ-tubulin depletion (Ori-McKenney et al., 2012). This was attributed to microtubule nucleation from γ-TuRCs at Golgi outposts (Ori-McKenney et al., 2012), but it was later shown that most Golgi outposts, including those positioned in the distal regions of the arbor, do not recruit detectable levels of γ-tubulin–GFP (Mukherjee et al., 2020). Based on our current data, we propose that γ-TuRCs are instead recruited via augmin to the sides of pre-existing microtubules. The role of augmin in supporting higher-order branching is also supported by a similar study published in this issue (Zhang et al., 2024). There are several non-mutually exclusive possibilities for how microtubules nucleated from augmin-bound γ-TuRCs might ultimately grow within terminal dendrites. First, augmin-bound γ-TuRCs could nucleate microtubules at the base of nascent branchpoints with the daughter microtubule growing directly into the nascent dendrite (Fig. 6A). Recruitment of augmin to the branchpoint could be stochastic, helping to explain why many nascent dendrites retract and disappear, or it could be influenced by the presence of other regulators of branching, such as Arp2/3 (Stürner et al., 2019). Second, augmin could amplify microtubules throughout the arbor, increasing the possibility that microtubules turn into nascent dendrites (Fig. 6B), although our EB1–GFP data shows that microtubule growth is actually increased in secondary and tertiary dendrites after augmin depletion. Finally, augmin might amplify anterograde microtubules already present within nascent dendrites (Fig. 6C). An alternative possibility is that augmin helps maintain correct microtubule polarity to ensure the proper delivery of material needed for new branching events (Fig. 6D). Future studies will be required to elucidate the exact molecular mechanism underlying the role of augmin in dendrite branching.

Intriguingly, we find that Grip71^NEDD1^ depletion results in similar phenotypes to those seen upon augmin depletion. However, both these phenotypes are weaker than those resulting from γ-TuRC depletion. First, this suggests that the γ-TuRC protein Grip71^NEDD1^ is not a general γ-TuRC targeting factor in class IV da neurons, which is similar to what occurs in dividing cells where Grip71^NEDD1^ is essential for γ-TuRC recruitment to spindle microtubules but has only a minor role in recruitment to centrosomes (Reschen et al., 2012; Vérollet et al., 2006; Zhu et al., 2023). Second, it is consistent with Grip7^NEDD1^ being required for augmin-mediated recruitment of γ-TuRCs in da neurons. This is interesting because the involvement of Grip7^NEDD1^ in augmin-mediated nucleation might influence the angle of the nucleation event. Depletion of NEDD1 from *Arabidopsis thaliana* results in a selective loss of branched nucleation events in cortical arrays, with the remaining events being parallel (i.e. having a 0˚ angle). The possibility that Grip7^NEDD1^ promotes angled nucleation events fits with an observed average angle of ∼36˚ in *Drosophila* S2 cells (Verma and Maresca, 2019), where Grip7^NEDD1^ is required for proper spindle assembly (Dobbelaere et al., 2008; Moutinho-Pereira et al., 2013; Vérollet et al., 2006). Whether Grip7^NEDD1^ promotes branched nucleation events in class IV neurons remains to be determined, but such events would allow daughter microtubules to grow directly into a nascent dendrite when nucleated at the branch point (Fig. 6A). Branched nucleation events occurring elsewhere might be accommodated if the daughter microtubule can bend within the constraints of the cell membrane. In cultured mammalian neurons and hippocampal tissue, however, NEDD1 is downregulated at later developmental stages, despite there still being an interaction between augmin and γ-TuRCs (Sánchez-Huertas et al., 2016). Thus, the finer details of augmin-mediated nucleation within neurons might differ between species.

An important question is how γ-TuRCs are employed to help regulate different aspects of class IV dendritic arbor development. Although γ-TuRCs appear to promote higher-order branching via the augmin pathway, the difference in phenotypes between grip91^GCP3^-RNAi and Dgt4-RNAi suggest that γ-TuRCs function independently of augmin to mediate the growth of primary and secondary dendrites and major branching events. It is possible that the γ-TuRCs associated with the few proximal Golgi outposts within primary branchpoints nucleate microtubules that contribute to these processes or that a putative MTOC observed close to the tip of extending primary dendrites (Yoong et al., 2020) recruits γ-TuRCs that ultimately promote the growth and branching of primary dendrites. Nevertheless, we cannot completely rule out that augmin is required for these major growth and branching events and that residual augmin left over after RNAi depletion is sufficient to mask any major defects.

Our conclusions on the requirements for γ-TuRCs and augmin were drawn in part from the analysis of dendritic arbor re-growth in pupae. Here, we found the phenotypes induced by RNAi expression to be stronger than those observed in larvae. This might be due to ineffective RNAi during early arbor development in embryos and larvae, possibly due to persistent maternal contributions of protein and protein complexes. Indeed, in our hands, *γ-tubulin23c*-null mutant flies can survive until late larval/early pupal stages, which is presumably only possible due to a persistent maternal contribution. Thus, future studies may consider analyzing phenotypes in pupae as well as embryos or larvae. For example, examining EB1–GFP comets in pupal neurons depleted for γ-TuRCs would be informative, as previous studies in larvae report relatively minor changes, or no changes, in EB1–GFP comet frequency after γ-tubulin depletion (Nguyen et al., 2014; Ori-McKenney et al., 2012). Moreover, one might see stronger effects on microtubule dynamics after augmin depletion. The use of other techniques to directly and quickly deplete or perturb proteins within neurons would also be of value, as was recently done for Kinesin-1 (Xu et al., 2023 preprint).

Although the importance of γ-TuRCs for the development of class IV da neuron arbors is now clear, γ-TuRC-independent microtubule nucleation might also play a role. As mentioned above, most Golgi outposts do not associate with detectable levels of γ-tubulin, yet EB1–GFP comets do grow out from them (Ori-McKenney et al., 2012; Zhou et al., 2014). Moreover, microtubule polarity is perturbed when Golgi outposts are mis-localized in axons even when γ-TuRC proteins have been depleted (Yang and Wildonger, 2020). It remains to be explored whether γ-TuRC-independent microtubule nucleation plays an important role in arbor development. Indeed, more studies are now required to fully elucidate the intricacies of how γ-TuRCs, augmin and γ-TuRC-independent microtubule nucleation events are regulated to ensure correct dendritic arbor growth and branching.

## MATERIALS AND METHODS

### Contact for reagent and resource sharing

Further information and requests for resources and reagents should be directed to and will be fulfilled by the author for correspondence.

### Fly stocks

All *Drosophila* stocks and crosses were maintained at 18 or 25°C on Iberian fly food made from dry active yeast, agar and organic pasta flour, supplemented with nipagin, propionic acid, pencillin-streptomycin and food coloring (per litre: nipagin, 20 ml; proprionic acid, 3.2ml; and pencillin-streptomycin, 8 ml). BL denotes stock numbers for stick from the Bloomington *Drosophila* stock center.

The following fluorescent alleles were used in this study: ppk-CD4-tdGFP (BL 35842), γ-tubulin23C-sfGFP (Tovey et al., 2018), γ-tubulin23c-eGFP (Mukherjee et al., 2020), UAS-EB1-GFP (BL 35512), UAS-mCD8-RFP (gift from Peter Lawrence and Jose Casal, Department of Zoology, University of Cambridge, UK). The following Gal4 and RNAi lines were used in this study: IT.Gal4-dgt5 (BL 77584), ppk-Gal4 (BL 32078), 221-Gal4 (BL 26259), UAS-IVS-myr-GFP (gift from Matthias Landgraf, Department of Zoology, University of Cambridge, UK), UAS-grip91^GCP3^-RNAi1 (VDRC 104667), UAS-grip91^GCP3^-RNAi2 (BL 31201), UAS-grip84^GCP2^-RNAi1 (VDRC 105640), UAS-grip84^GCP2^-RNAi2 (BL 33548), γ-tubulin23C-RNAi1 (BL 31204), γ-tubulin23C-RNAi2 (VDRC 19130), UAS-Dicer 2 (BL 24646), UAS-grip7^NEDD1^-RNAi1 (VDRC v31228), UAS-grip7^NEDD1^-RNAi2 (NIG 10346R-1), UAS-dgt2-RNAi1 (BL31729), UAS-dgt2-RNAi2 (v330504), UAS-dgt3-RNAi1 (VDRC 103980), UAS-dgt3-RNAi2 (BL58137), UAS-dgt4-RNAi (VDRC 108969), UAS-dgt5-RNAi (BL60366). UAS-γ-tubulin37C-RNAi (BL32513) was used as the control in all cases. We confirmed by PCR that any RNAi line from the VDRC KK library did not contain a known aberrant insertion at genomic position 40D (data not shown; this information is now available on the VDRC website anyway), which upregulates the Hippo pathway and is mistakenly present in some VDRC KK library constructs (see information on VDRC website).

For examining the morphology of pupal or larval class IV neurons, we used flies expressing one copy of ppk-CD4-tdGFP and containing one copy of UAS-Dicer2 and one copy of the appropriate UAS-driven RNAi line expressed under the control of one copy of ppk-Gal4. For examining the morphology of larval class I neurons, we used flies containing one copy of UAS-mCD8-RFP, one copy of UAS-Dicer2, and one copy of the appropriate UAS-driven RNAi line expressed under the control of one copy of 221-Gal4. For examining the endogenous localization of γ-tubulin23C, we used flies expressing γ-tubulin23C-sfGFP and γ-tubulin23C-eGFP (i.e. 2 copies of γ-tubulin23C-GFP). For examining the localization of γ-tubulin23C during Grip91^GCP3^ depletion, we used flies expressing two copies of γ-tubulin23C-(sf/e)GFP (as above) in combination with one copy of UAS-grip91^GCP3^ RNAi and UAS-Dicer2 expressed under the control of one copy of 221-Gal4. For examining microtubule dynamics in control-RNAi or dgt4-RNAi neurons, we used flies containing one copy of UAS-EB1-GFP, one copy of UAS-Dicer 2 and one copy of either UAS-γ-tubulin37C-RNAi or UAS-dgt4-RNAi expressed under the control of one copy of ppk-Gal4. For examining *dgt5* expression, we used flies containing one copy of IT.Gal4-dgt5 and one copy of UAS-IVS-myr-GFP.

### Antibodies

The following primary antibodies were used: anti-GFP mouse monoclonal at 1:250 (Roche, 11814460001), Alexa-Fluor-647-conjugated anti-HRP goat polyclonal at 1:500 (Jackson, AB_2338967), and anti-GM130 rabbit polyclonal at 1:300 (Abcam, ab32337). The following secondary antibodies were used: Alexa Fluor 488 goat anti-mouse-IgG at 1:500 (Thermo Fisher Scientific, 10138324) and Alexa Fluor 568 anti-rabbit-IgG at 1:500 (Thermo Fisher Scientific, 10463022).

### Immunostaining and fixed cell imaging

Dissected larvae were processed as previously described (Mukherjee et al., 2020). Briefly, fillet preparations were fixed in freshly prepared 4% formaldehyde for 20 min at room temperature and were then washed four times for 10 min in PBS plus 0.1% Triton X-100 (PBST). Blocking was carried out in PBST plus 5% BSA for 1 h at room temperature. Preparations were incubated with appropriate primary antibodies diluted in PBST overnight at 4°C. After washing in PBST for ∼8 h, changing washes every 30–45 min, samples were incubated in secondary antibodies diluted in PBST overnight at 4°C. The fillet preparations were then washed for ∼8 h, changing washes every 30–45 min in PBST before mounting in Mowiol. They were stored at −20°C and imaged within a week. Neurons within segments A2 to A6 were imaged. Imaging of γ-tubulin–GFP within the soma was carried out using an Olympus FV3000 scanning inverted confocal system run by FV-OSR software with a 60×1.4NA silicone immersion lens (UPLSAPO60xSilicone). Images of neuronal somas within IT.Gal4-dgt5>UAS-IVS-myr-GFP flies were collected on a Zeiss Axio Observer.Z1 inverted CSU-X1 Yokogowa spinning disk system with 2 ORCA Fusion camera (Hamamatsu) run by Zeiss Zen2 acquisition software using a 60×1.4NA oil immersion lens (Zeiss). *Z*-stacks with 0.5 µm spacing were acquired to cover all neuronal soma.

### Live imaging

All imaging was carried out at ambient room temperature (∼21°C). Imaging of overall neuronal morphology was carried out on a Leica SP5 point scanning upright confocal system run by LAS AF software with a 20×0.7NA multi-immersion lens (11506191) with glycerol as the immersion medium. *Z*-stacks with 1 µm spacing were acquired to cover the whole arbor. For imaging class IV v'ada neurons within pupae, white pre-pupae (0 h APF) were collected and placed in a sealed Petri dish with some moistened tissue paper and aged for the required duration in a 25°C incubator. The staged pupae were stuck on double-sided sticky tape and removed from their pupal casing. The pupae were mounted on a glass slide with a small amount of halocarbon oil and a coverglass was placed gently on top of the pupae using dental wax feet as spacers. The mounted pupae were imaged immediately. For larval neuron morphology, newly hatched (within 1 h) larvae were collected and placed on a new plate to be staged appropriately at 25°C. The larvae were then placed in a drop of glycerol and gently flattened between a slide and a 22×22 mm coverslip, held in place by tape, and imaged immediately. Imaging of EB1–GFP comets and the dynamic morphology of terminal dendrites was carried out on a Leica DMi8 inverted wide-field microscope controlled by µManager software and coupled to a BSI Prime Express monochrome camera (QImaging) and a CoolLED pE-300 Ultra light source using a 63×1.3NA oil objective (Leica 11506384). Wandering third-instar larvae (between ∼96 h and ∼120 h AEL) were placed in a drop of glycerol and gently held between a slide and a 22×22 mm coverslip using tape to hold down the coverslip, and imaged immediately. Care was taken not to flatten the larvae too much, as we found that fully squashing the larvae resulted in abnormal neuronal morphology defects. For EB1–GFP, five *Z*-stacks of 0.5 µm spacing were acquired every 5 s. For analyzing the dynamic morphology of terminal dendrites, ten Z-stacks of 1 µm spacing were acquired every 5 min for a total of 30 min.

### Image analysis and statistics

All images were processed in Fiji software (ImageJ). Images of neuronal morphology, γ-tubulin–GFP within soma, the *dgt5* Gal4 trap, and the dynamic morphology of terminal dendrites are maximum intensity projections of *Z*-stacks. To determine peripheral area for a neuron, a line was manually drawn around the neuronal field by joining the tips of dendrites at the edge of the arbor. In larvae, care was taken not to draw the line within large empty regions between dendrites in order to better measure the outgrowth of primary and secondary dendrites. Dendrite tips were counted manually for each neuron. To measure the length of the longest primary dendrite in pupae, a line was manually drawn along the dendrite and its length measured in Fiji software. To measure the total length of the primary and secondary dendrites in pupae, segmented lines were manually drawn along the dendrites and their lengths measured in Fiji and then summed. Amira software was used to measure the total arbor length and tip number of class I da neurons.

For the analysis of the γ-tubulin–GFP signal at somatic Golgi stacks in control versus grip91^GCP3^-RNAi neurons, ROIs were drawn around individual Golgi stacks and their sum fluorescence intensities were background corrected using a mean cytosolic intensity measurement. EB1 comets were manually scored for their number and direction within different types of dendrites. Data was processed in Microsoft Excel. Statistical analysis and graph production were performed using GraphPad Prism. For analysing the expression of IT.Gal4-dgt5>UAS-IVS-myr-GFP, intensity measurements within the soma were used to determine whether the expression was similar, higher or lower between class I and class IV da neurons.

When parametric tests were used, tests for normality were first performed using D'agostino and Pearson tests, Anderson–Darling tests, Shapiro–Wilk tests and Kolmogorov–Smirnov tests. Datasets were considered to be normally distributed when at least one test passed the normality test. All *t*-tests were two-sided. When using one-way ANOVA we assumed equal s.d. We corrected for multiple comparisons using Šídák hypothesis testing and multiplicity corrected *P*-values for each comparison were reported. When using Kruskal–Wallis tests, we corrected for multiple comparisons using Dunn's hypothesis testing and multiplicity corrected *P*-values for each comparison were reported. The following tests were performed to make comparisons between datasets: For Figs 1C,D and 3B–D we used a one-way ANOVA with correction for multiple comparisons. For Figs 1E,F,H, 2B,D, 4B,D,E, 5B,D, we used we used two-tailed unpaired *t*-tests. For Fig. 1G, we used a Kolmogorov–Smirnov test. For Fig. 3F, we used a Kruskal–Wallis test. For Fig. 4F we used a Chi-squared test. For Fig. 3D,E,G, we used one-way ANOVA. For Fig. 4G,J we used Fisher's exact tests. For Fig. 4I we used a Mann–Whitney test. The data in Fig. S3G was determined to be log-normally distributed and so the data was first log transformed by taking the log_10_ values before performing a two-tailed unpaired *t*-test.

### RT-qPCR

For each of three biological replicates, wing imaginal discs were dissected from 25 males flies then washed with phosphatate-buffered saline (PBS) and stored at −70°C in Trizol (Thermo Fisher Scientific) until use. Total RNA was isolated from the sample using RNeasy Mini Kit (Qiagen). cDNA was synthesized using Superscript III Reverse Transcription Kit (Invitrogen). cDNA synthesis for each biological replicate was repeated three times on three separate days to generate nine samples for quantitative RT-PCR. Quantitative RT-PCR was run in triplicate for each of the nine samples in 15 µl reactions in a real-time PCR system (LightCycler^®^ 480 Multiwell Plate 384, Roche, Life Science) using SYBR-Green qPCR mix (Roche, Life Science). qPCR software was used for analyzing cycle threshold (Ct) values. Fold change in mRNA levels (expressed as 2^−ΔΔC^) was normalized to the mean expression of four housekeeping genes: α-tubulin84b, Ribosomal Protein S2 (SOP), Ribosomal Protein L32 (RPL32) and Glyceraldehyde 3-phosphate dehydrogenase (GAPDH-2). The sequence of the primers used is given in Table S3. Table S4 contains all the raw data.

## Supplementary Material



10.1242/joces.261534_sup1 Supplementary information

Table S4. Raw data for RT-qPCR.
