# Supplementary material for: γ-TuRCs and the augmin complex are required for the development of highly branched dendritic arbors in Drosophila
Source: J Cell Sci. 2024 May 10;137(9):jcs261534. doi: 10.1242/jcs.261534 (PMC11128279; doi:10.1242/jcs.261534)
Supplement: Supplementary information [file joces-137-261534-s1.pdf]

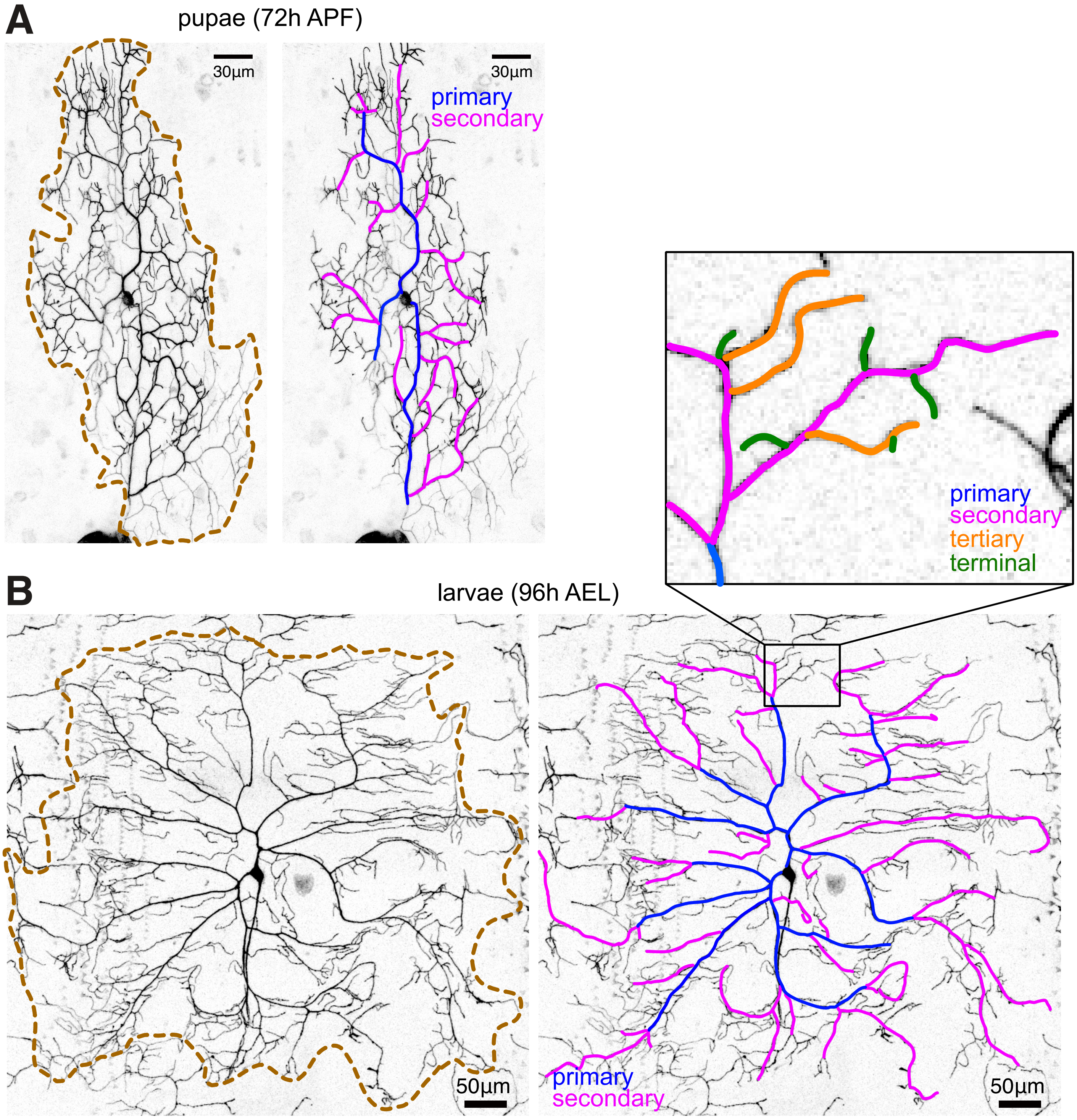

Fig. S1. Images to show which dendrites were identified as primary, secondary, tertiary and terminal dendrites. (A) Confocal images show the same pupal class IV v'ada neuron expressing CD4-tdGFP at 72h APF. The image on the right is overlaid with lines indicating primary (blue) and secondary (magenta) dendrites. The distinction between primary and secondary dendrites was made based on the thickness and length of the dendrites and on the angle at which the dendrites emerge from the branchpoint. **(B)** Confocal images show the same larval class IV ddaC neuron expressing CD4-tdGFP at 76h AEL. The image on the right is overlaid with lines indicating primary (blue), secondary (magenta), tertiary (orange), and terminal (green) dendrites. The distinction was made based on the thickness and length of the dendrites and the angle at which the dendrites emerge from the branchpoint.

Journal of Cell Science • Supplementary information

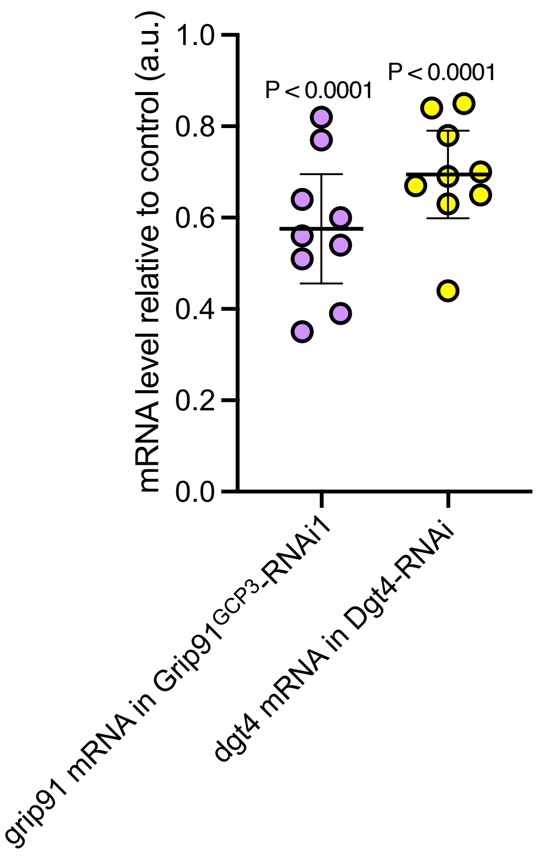

Fig. S2. RT-qPCR analysis of wing disks expressing either γ-TuRC-RNAi or Augmin-RNAi. Graph shows mRNA levels relative to control estimated using quantitative RT-PCR for grip91 and dgt4 in imaginal discs expressing grip91-RNAi and dgt4-RNAi, respectively. For each condition, 3 biological replicate samples (each 50 discs from 25 flies) of mRNA were prepared. For each biological replicate, 3 repeats of cDNA preparation and subsequent qPCR analysis were performed to generate the 9 values that appear on the graph. Each repeat was normalised to a control value of 1 obtained from discs of w1118 flies by using values from 4 housekeeping genes. The list of genes and the sequences of the primers used are shown in Table S3. Raw data can be found in Source data file 1. The results are presented as the mean 95% CIs. One-sample t-tests were used to test for a significance difference from 1. P values are shown above each dataset.

Journal of Cell Science • Supplementary information

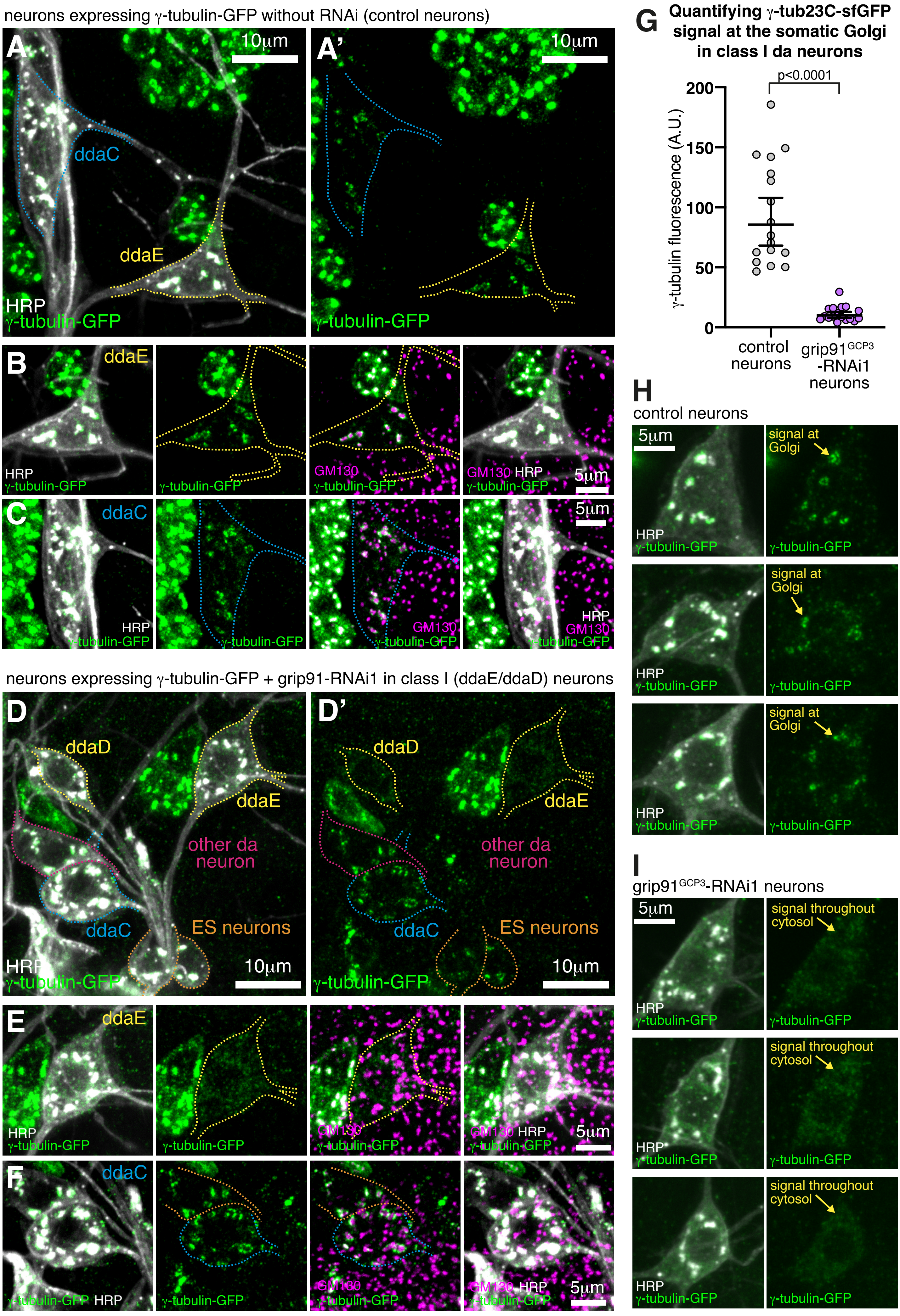

Journal of Cell Science • Supplementary information

Fig. S3. Depletion of Grip91^{GCP3} results in loss of γ-tubulin-GFP from somatic Golgi stacks. (A-F) Confocal images show the somas of sensory neurons within the dorsal cluster of 3rd instar larva expressing endogenously-tagged γ-tubulin-GFP alone (A-C) or γ-tubulin-GFP and UAS-grip91^{GCP3}-RNAi (expressed by 221-Gal4 specifically within class I ddaD and ddaE neurons) (D-F) and immunostained for GFP (green), HRP (marking membranes including Golgi stacks, greyscale), and GM130 (marking cis-Golgi, magenta). (A, A') and (D, D') show a wide view of the dorsal clusters, with (A) and (D) showing the overlay of the γ-tubulin-GFP and HRP signals and (A') and (D') showing just the γ-tubulin-GFP signal. (B), (C), (E), and (F) display class I ddaE neurons (B,E), which express the RNAi constructs, and class IV ddaC neurons (C,F), which do not express the RNAi constructs. Note how γ-tubulin-GFP associates with HRP and GM130 in da neurons under normal conditions (A-C) but does not associate with HRP and GM130 in class I da neurons when grip91^{GCP3}-RNAi is expressed (D,E). Note also how γ-tubulin-GFP still associates with Golgi within both other da and ES neurons that do not express grip91^{GCP3}-RNAi (D,F). Scale bars are indicated within the images. (**G**) Graph shows fluorescence intensity measurements of somatic Golgi stacks within either class I da neurons expressing γ-tubulin-GFP alone (grey, n=218 Golgi stacks from 17 neurons) or class I da neurons expressing both γ-tubulin-GFP and grip91^{GCP3}-RNAi (red, n=249 Golgi stacks from 17 neurons). $P<0.0001$, unpaired two-sided t-test on $\log_{10}$ values. (**H, I**) Images showing examples of class I ddaE neuron soma from 3rd instar larva expressing endogenously-tagged γ-tubulin-GFP alone (H, control neurons) or γ-tubulin-GFP and UAS-grip91^{GCP3}-RNAi (I, grip91^{GCP3}-RNAi neurons) and immunostained for GFP (green) and HRP (greyscale). Note how the γ-tubulin-GFP signal is predominantly concentrated at Golgi stacks in control neurons but is dispersed throughout the cytosol in grip91^{GCP3}-RNAi neurons.

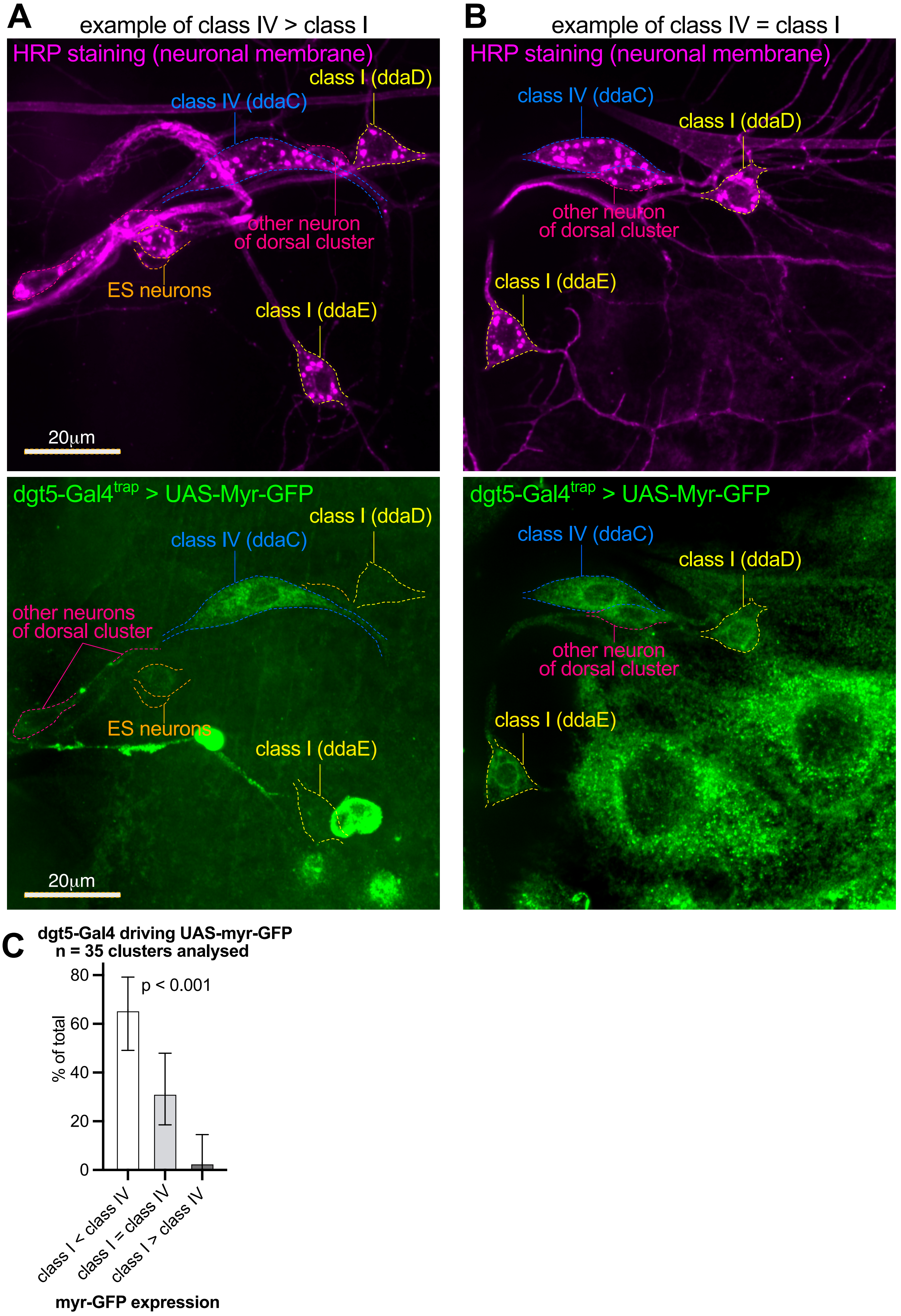
A
example of class IV > class I
HRP staining (neuronal membrane)
class I (ddaD)
class IV (ddaC)
other neuron
of dorsal cluster
ES neurons
class I (ddaE)
20μm
dgt5-Gal4trap > UAS-Myr-GFP
class I (ddaD)
class IV (ddaC)
other neurons
of dorsal cluster
ES neurons
class I (ddaE)
20μm
B
example of class IV = class I
HRP staining (neuronal membrane)
class IV (ddaC)
class I (ddaD)
other neuron
of dorsal cluster
class I (ddaE)
dgt5-Gal4trap > UAS-Myr-GFP
class IV (ddaC)
class I (ddaD)
other neuron
of dorsal cluster
class I (ddaE)
C
dgt5-Gal4 driving UAS-myr-GFP
n = 35 clusters analysed
p < 0.001
% of total
80
60
40
20
0
class I < class IV
class I = class IV
class I > class IV
myr-GFP expression

Fig. S4. The Augmin component Dgt5 is expressed at higher levels in class IV neurons compared to class I neurons. (A,B) Confocal images show the somas of sensory neurons within the dorsal cluster of 3rd instar larva expressing UAS-Myr-GFP (green) under the control of a dgt5-Gal4^{trap} and immunostained for HRP (marking neuronal membranes, magenta). Yellow dotted lines on the dgt5-Gal4^{trap} images show the outlines of the neuronal somas identified with HRP staining. Based on neuronal morphology and position within the cluster, class I ddaE and ddaD, class IV ddaC, and external sensory neurons could be identified and are indicated. The panels on the left and right show examples of where dgt5-Gal4^{trap} expression is higher in the class IV ddaC neuron compared to the class I neurons or roughly equal, respectively. **(C)** Graph quantifying the frequency of observed differences or similarities in expression levels of dgt5-Gal4^{trap} between class I and class IV neurons from 35 clusters. Note that expression is normally higher in class IV neurons than class I neurons.

Table S1. RNA lines used for the essential γ-TuRC components.

lab code	gene	RNAi type	stock no.	40D present
R45	g-tub37C	TRiP valium 20	BL32513	n/a
R1	g-tub23C RNAi1	TRiP valium 1	BL31204	n/a
R21	g-tub23C RNAi2	VDRC GD	v19130	n/a
R33	grip84^{GCP2} RNAi1	VDRC KK	v105640	no
R41	grip84^{GCP2} RNAi2	TRiP valium 20	BL33548	n/a
R34	grip91^{GCP3} RNAi1	VDRC KK	v104667	no
R42	grip91^{GCP3} RNAi2	TRiP valium 1	BL31201	n/a

Table S2. RNAi lines used against various augmin components.

lab code	gene	RNAi type	stock no.	40D present
R65	grip71^{NEDD1} RNAi1	VDRC GD	v31228	n/a
R74	grip71^{NEDD1} RNAi2	NIG	10346R-1	n/a
R64	dgt2 RNAi1	TRiP valium 1	BL31729	n/a
R55	dgt2 RNAi2	VDRC TRiP valium 20	v330504	n/a
R68	dgt3 RNAi1	VDRC KK	103980	no
R62	dgt3 RNAi2	VDRC TRiP valium 20	BL58137	n/a
R68	dgt4 RNAi	VDRC KK	v108969	no
R63	dgt5 RNAi	VDRC TRiP valium 20	BL60366	n/a

Table S3. Primer sequences.

Gene	Forward primer (5'->3')	Reverse primer (5'->3')
a-tub84b	CACACCACCCTGGAGCATTC	CCAATCAGACGGTTCAGGTTG
SOP	CACCCCAATAAAGTTGATAGACCT	ACCACCACGAGAGCCAAAT
RPL32	CTTCATCCGCCACCAGTC	CGACGCACTCTGTTGTCG
GAPDH	GATGCTCCCATGTTCGTTTGC	GTCCATCACGCCACAACTTG
dgt4	CGCCATCATTGACAACTGAGG	GCTGTCTTCCTGAAAACGGC
grip91	TGTGGCAACCAATTCCACTGA	TTTTCGTGAACTTCGGGCATC

Table S4. Raw data for RT-qPCR.

Available for download at
https://journals.biologists.com/jcs/article-lookup/doi/10.1242/jcs.261534#supplementary-data

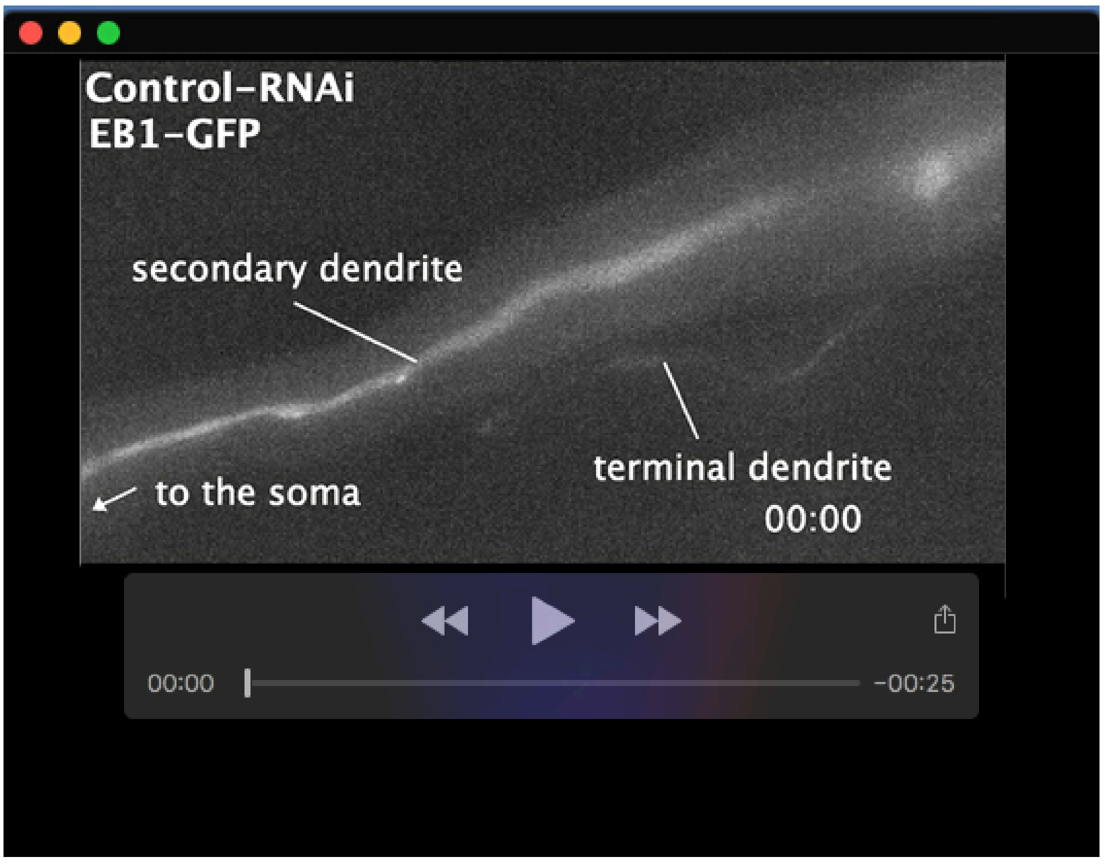

Movie 1. Movie showing EB1-GFP comets within the distal region of control-RNAi neurons. Comets can be seen travelling towards the soma (retrograde) in the secondary dendrite and away from the soma in the terminal dendrite. Movie plays at 10 fps.

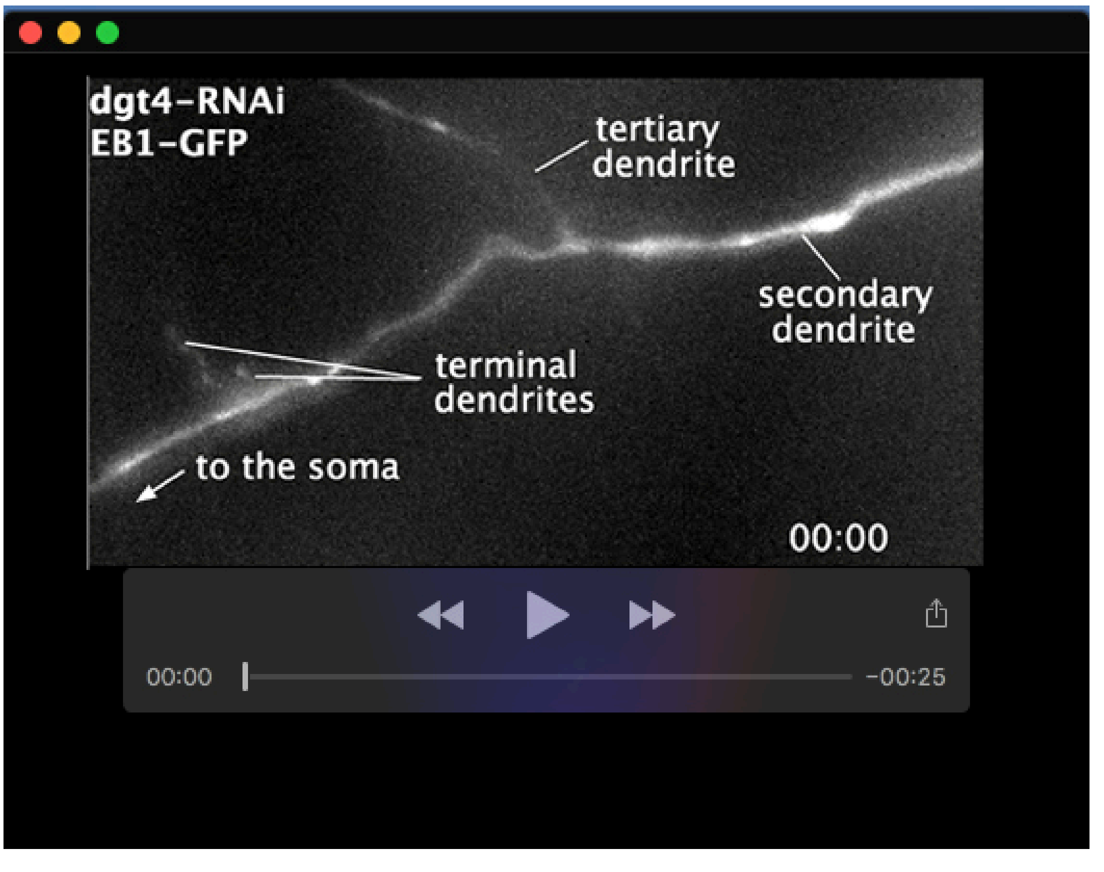

Movie 2. Movie showing EB1-GFP comets within the distal region of Augmin-RNAi neurons. Comets can be seen travelling both towards the soma (retrograde) and away from the soma (anterograde) in the secondary dendrite. Two terminal dendrites can be observed – an EB1-GFP comet travels within one terminal dendrite but no EB1-GFP comets travel in the other terminal dendrite. Movie plays at 10 fps.

Journal of Cell Science • Supplementary information

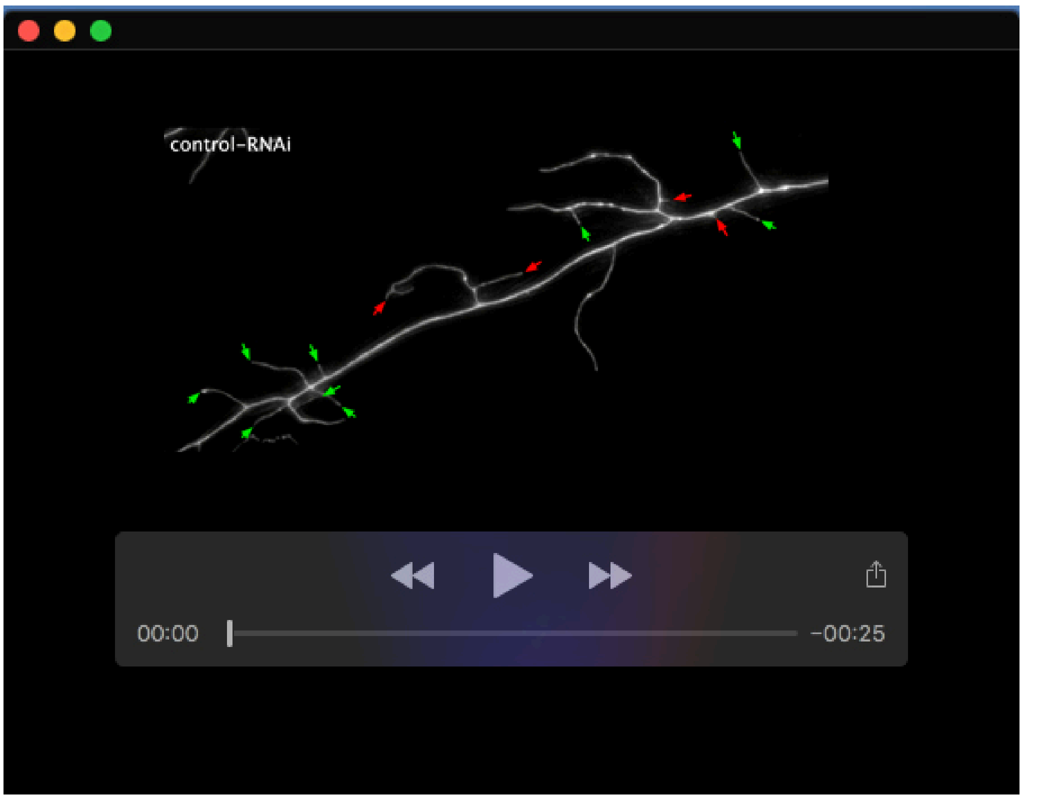

Movie 3. Movie showing terminal dendrite dynamics within the distal region of a control-RNAi neuron. Distal region of a class IV da neuron expressing ppk-CD4-tdGFP and control-RNAi. Green arrows indicate terminal dendrites that are present at the beginning and the end of the movie. Red arrows indicate terminal dendrites that are present at the beginning of the movie but have disappeared by the end. Blue arrows indicate terminal dendrites that are absent at the beginning of the movie but appear during the movie. Movie plays at 5 fps.

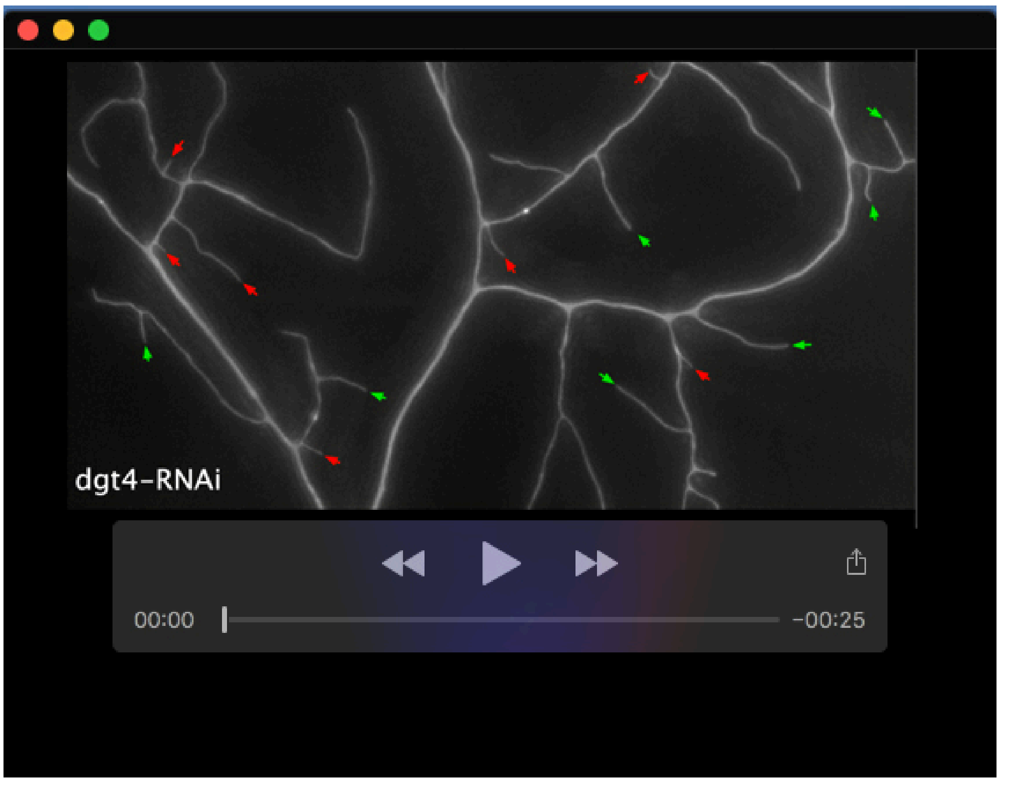

Movie 4. Movie showing terminal dendrite dynamics within the distal region of a Augmin-RNAi neuron. Distal region of a class IV da neuron expressing ppk-CD4-tdGFP and Augmin-RNAi. Green arrows indicate terminal dendrites that are present at the beginning and the end of the movie. Red arrows indicate terminal dendrites that are present at the beginning of the movie but have disappeared by the end. Blue arrows indicate terminal dendrites that are absent at the beginning of the movie but appear during the movie. Movie plays at 5 fps.

Journal of Cell Science • Supplementary information
