# Supplementary material for: γ-TuRCs and the augmin complex are required for the development of highly branched dendritic arbors in Drosophila
Source: J Cell Sci. 2024 May 10;137(9):jcs261534. doi: 10.1242/jcs.261534 (PMC11128279; doi:10.1242/jcs.261534)
Supplement: Supplementary file 1 [file joces-137-261534_review_history.pdf]

Journal of Cell Science

γ-TuRCs and augmin are required for the development of highly branched dendritic arbors in *Drosophila*

Amrita Mukherjee, Yaiza Andrés Jeske, Isabelle Becam, Anaelle Taïeb, Paul Brooks, Joanna Aouad, Clementine Monguillon and Paul J. Conduit
DOI: 10.1242/jcs.261534

Editor: Michael Way

Review timeline

Original submission:	4 August 2023
Editorial decision:	30 August 2023
First revision received:	29 February 2024
Editorial decision:	3 April 2024
Second revision received:	4 April 2024
Accepted:	5 April 2024

Original submission

First decision letter

MS ID#: JOCES/2023/261534

MS TITLE: γ-TuRCs and Augmin are required for the development of highly branched dendritic arbors in Drosophila

AUTHORS: Amrita Mukherjee, Yaiza Andres Jeske, Isabelle Becam, Paul Brooks, joanna aouad, clementine monguillon, and Paul J Conduit

We have now reached a decision on the above manuscript.

To see the reviewers' reports and a copy of this decision letter, please go to: https://submit-jcs.biologists.org and click on the 'Manuscripts with Decisions' queue in the Author Area. (Corresponding author only has access to reviews.)

As you will see, the reviewers raise a number of substantial criticisms that prevent me from accepting the paper at this stage. They suggest, however, that a revised version might prove acceptable, if you can address their concerns. If you think that you can deal satisfactorily with the criticisms on revision, I would be pleased to see a revised manuscript. We would then return it to the reviewers.

Please ensure that you clearly highlight all changes made in the revised manuscript. Please avoid using 'Tracked changes' in Word files as these are lost in PDF conversion.

I should be grateful if you would also provide a point-by-point response detailing how you have dealt with the points raised by the reviewers in the 'Response to Reviewers' box. Please attend to all of the reviewers' comments. If you do not agree with any of their criticisms or suggestions please explain clearly why this is so.

Reviewer 1

© 2024. Published by The Company of Biologists under the terms of the Creative Commons Attribution License (https://creativecommons.org/licenses/by/4.0/).

Advance summary and potential significance to field

The gamma-tubulin ring complex, or gamma-TURC, is an important microtubule nucleator in cells and exists both in and outside of canonical centrosomes. As the best characterized microtubule nucleator, gamma-TURC is proposed to play crucial roles in all or most processes that involve microtubule growth. Somewhat surprisingly, gamma-tubulin depletion was previously shown to only cause mild defects in higher order dendrite elaboration of Drosophila peripheral sensory neurons. In this manuscript, Mukherjee et al re-investigate the role of the gamma-TURC as well as that of HAUS/Augmin, a related microtubule-regulating complex. They show that gamma-TURC depletion causes stronger defects at later developmental stages, possibly indicating that previous results had been blurred by maternal contribution and protein perdurance. They further show that HAUS/Augmin depletion causes similar, but milder defects than that of gamma-TURC and speculate that it may be involved in a subset of gamma-TURC functions.

The mechanisms of microtubule nucleation and organization in neurites are of high importance to our understanding of neuronal cell biology but largely still unresolved. Whether gamma-TURC is a major factor has been debated, especially the Drosophila data have been inconclusive. This manuscript attempts a clarification by looking at another developmental period where analysis may be more conclusive. The solid, albeit somewhat superficial, analysis demonstrates an important role for gamma-TURC and its effector HAUS but also clearly shows other mechanisms must be at play as well. I have a few technical comments, and it would be nice to deepen the link between and HAUS/Augmin and microtubules a bit more.

Comments for the author

Major comments

1. The conclusion that gamma-TURC is a major nucleator in sensory neurons is based on the strong phenotype caused by a single RNAi construct against Grip91, all other RNAi lines cause milder defects. What is the evidence that this is not an off-target effect? The authors could address this by using another manipulation (e. g., in Ori-McKenney et al., 2012, the effect of gamma-tub23C RNAi is enhanced by crossing in a gamma-tub23C mutant allele).

2. The authors should try to tie the gamma-TURC phenotype at the pupal stage to microtubules, e. g., by measuring the microtubule density (using EB1GFP or similar).

3. To ascertain that HAUS/Augmin acts via its canonical gamma-TURC based mechanism, the authors should address the genetic relationship between the two complexes. Does co-depletion of gamma-TURC and HAUS leads to alterations of the dendrite defects that can be interpreted? Or does gamma-tub overexpression suppress Augmin LOF?

4. Localization of augmin would be interesting to know. But not a must.

5. Do higher order dendrite fail to form in Augmin LOF? Or do they form and retract? Could be addressed in a timelapse experiment

6. Figs. 1E, F. Does the ratio - branchpoints per um – differ?

Minor comments

1. Some references are incorrect. References for PNS neuron pruning: Kuo, Jan & Jan 2005, Williams & Truman 2005, but not Williams and Shepherd 1999. First reference describing dendrite regrowth: Kuo, Jan & Jan 2005

2. Line 150 “Although da neurons are rarely analysed in pupae…“ is a bit off. Though not cited here, there are a number of papers that look specifically at this stage to study dendrite regenerative and/or growth mechanisms

© 2024. Published by The Company of Biologists under the terms of the Creative Commons Attribution License (https://creativecommons.org/licenses/by/4.0/).

3. Figs 1F, 3E. Can the data for the different genotypes be displayed next to each other? Above each other, the data is harder to interpret. Also please add more percentage points on the y axis

Reviewer 2

Advance summary and potential significance to field

In this manuscript Mukherjee and colleges address the role of Augmin and gamma-tubulin in Drosophila dendrite development. Although Augmin function in neurons has been described before (mainly) in cultured mammalian neurons, addressing its role in another system and importantly in vivo is relevant.

Comments for the author

Here the authors found that gamma-tubulin is more broadly required during development whereas Augmin is mainly required for the fine/distal branching. As Augmin is needed to nucleate microtubules from existing microtubules they looked at microtubule polarity and found some subtle defects, a bit surprisingly mainly in a more proximal branch.

In general the work is well performed. However I think that the authors should take it one step further to try to link the morphological defects to the microtubule defects. e.g. is microtubule density affected in the terminal branches, where does Augmin localize. Now the authors make the point that there are fewer terminal branches with EBs, however this is a very small effect and not significant, so not convincing this is a real effect of KD.

One other point that needs revision are the graphs in 1F and 3E. This is not the correct representation, e.g. in 3E I don't know if the Dgt4 in bin 2 should be read as 80% or as 30%. Either these need to be separated (like e.g. 4F) or the data should differently represented such that 1 condition goes to 100% (so column 1 control, column 2 KD1, …). Also the statistics are missing for this data.

Textual
I appreciate the efforts of the authors to make the text accessible to non-drosophila readers. However this could still be improved a bit, e.g. the testing of the KK library can maybe be placed in the methods and along the text I forgot that Grip71 = NEDD1

line 320 "these data … secondary dendrites" I don't follow how this is inferred from the results describe above which are quantifications of the terminal branching.

Line 343 It would make it easier for the reader to only describe the MT polarity as xx % anterograde (or retrograde) in the paragraph

Line 392 what is the "wac mutant"?

Line 393-398 : "In control … Augmin depletion" I think the point does not come across well. And lost the detailed comparison

Reviewer 3

Advance summary and potential significance to field

Neurons have elaborate dendritic arbors. These are arbors are microtubule-rich raising the question of how the microtubule cytoskeleton might influence dendrite morphogenesis. In this manuscript, the authors focus on the role that the nucleation of new microtubules by augmin and gamma-TuRC components might play in dendrite branch formation and, ultimately, shaping arbor structure. While both augmin and gamma-TuRC have been studied for many years, there has been the outstanding question of whether the fairly mild (or lack of) neuronal phenotypes may reflect an

© 2024. Published by The Company of Biologists under the terms of the Creative Commons Attribution License (https://creativecommons.org/licenses/by/4.0/).

incomplete reduction of their activity. A strength of this current study is the use of a dendrite re-growth assay during late stages of development to uncover any latent defects in dendrite morphogenesis. This manuscript nicely presents the dendrite morphogenesis phenotypes that result from knocking-down components of the augmin and gamma-TuRC complexes (it is a nice descriptive study). Overall, this is a very nice body of work, with just a few major concerns.

Comments for the author

1. The authors describe the use of the dendrite re-growth assay during pupal stages as an advantageous model to maximize the effects of RNAi-mediated knockdown (and depletion of any maternal contribution). But it is not clear whether the RNAi strains effectively knockdown protein levels. Can the authors assay the efficacy of the different RNAi strains to deplete the target proteins? For example, perhaps it is possible to use a broadly expressed Gal4 (such as elav-Gal4, or tub-Gal4) to express the RNAi constructs and then use western blots to assay the degree of knockdown. Doing this analysis at larval and pupal stages would potentially bolster the author's model that dendrite re-growth is affected because protein levels are more reduced during pupal v. larval stages.

2. What is known about the expression and distribution of augmin and gamma-TuRC components in the class IV and class I da neurons? It would be helpful to include information about the expression (and potentially localization) of these components. For example, are augmin components not well-expressed in class I neurons? Do augmin complex members localize to dendrites?

3. Is it possible to visualize gamma-Tub23C-GFP in dendrites and determine if its localization is disrupted by the knockdown of Grip91? This would support the authors' model that augmin is recruiting gamma-Tub/gamma-TuRC to positions of nascent branches in the arbor.

4. On a related note: The authors claim the "Augmin microtubule nucleation pathway as being the γ-TuRC-dependent…" (line 164), but no support for this is included in the manuscript. Is it possible to enhance the augmin phenotypes by reducing gamma-TuRC activity?

Minor Comments

5. For clarity, consider referring to Grip91-RNAi1 and Dgt4-RNAi as such throughout rather than switching to referring to them as "γ-TuRC-RNAi" and "Augmin-RNAi," respectively.

6. Please include some representative images for the data represented in Figure 4H, in particular the distal terminal dendrites.

7. Line 227: Change "the authors" to "others."

8. Line 445: Typo, change "forth" to "fourth."

9. Line 446: Some part of the sentence must have been inadvertently deleted ("possibility is does not depend on microtubule growth…"); please remedy.

10. Line 489-490: Yang and Wildonger (2020) reference should be removed here;
don't believe that it shows microtubule growth originating at Golgi outposts (?).

11. Figure 6D: The figure legend should clarify that this model represents "distal secondary branches" in the dendritic arbor; microtubule polarity was not affected in other parts of the arbor.

First revision

<u>Author response to reviewers' comments</u>

Dear Reviewers and Editors,

© 2024. Published by The Company of Biologists under the terms of the Creative Commons Attribution License (https://creativecommons.org/licenses/by/4.0/).

We thank the Reviewers for taking the time to provide constructive feedback for our paper. We have addressed the majority of your concerns and feel they significantly improve the paper. We apologise for not getting this resubmission back to you sooner.

Below, the Reviewer comments are in blue and our responses are in black.
Summary of the main additions/changes:

- GENE DEPLETION: We have performed RT-qPCR to show that the Grip91-RNAi and Dgt4-RNAi constructs are capable of depleting their respective gene products.
- MICROTUBULE DEFECTS: We have imaged and analysed more class IV da neurons expressing EB1-GFP so that we can now link the morphological defects produced by Augmin-RNAi to defects in microtubules. Importantly, we find that the frequency of EB1-GFP comets within terminal dendrites is significantly lower in Augmin-RNAi neurons compared to controls. This also gives us the statistical power to show that microtubule polarity is affected in distally located secondary and tertiary dendrites.
- DENDRITE DYNAMICS: We have also imaged class IV neurons through time to monitor the stability of terminal dendrites and better link morphology and microtubule defects. We found that terminal dendrites form at the same rate in Augmin-RNAi neurons compared to controls, but that they are significantly less stable (disappearing more frequently). This is consistent with reduced EB1-GFP comets in terminal dendrites (see point above), as microtubule growth within terminal dendrites has been shown to correlate with nascent dendrite stability (Ori-McKenney et al., 2012).
- AUGMIN EXPRESSION: We have used a Dgt5-Gal4-trap line to show that expression of Augmin components is higher in class IV da neurons compared to class I da neurons. This fits with the conclusion that Augmin promotes higher-order branching (which is lacking in class I neurons) and that Augmin depletion from class I da neurons has little effect on their morphology.
- DENDRITE LENGTH / BRANHPOINT RATIO: We have re-analysed the pupal neurons by measuring the combined length of primary and secondary branches and the number of primary and secondary branchpoints to allow us to record the ratio between major dendrite length and major branching events under different conditions.

Reviewer 1 Advance Summary and Potential Significance to Field:
The gamma-tubulin ring complex, or gamma-TURC, is an important microtubule nucleator in cells and exists both in and outside of canonical centrosomes. As the best characterized microtubule nucleator, gamma-TURC is proposed to play crucial roles in all or most processes that involve microtubule growth. Somewhat surprisingly, gamma-tubulin depletion was previously shown to only cause mild defects in higher order dendrite elaboration of Drosophila peripheral sensory neurons. In this manuscript, Mukherjee et al re-investigate the role of the gamma-TURC as well as that of HAUS/Augmin, a related microtubule-regulating complex. They show that gamma-TURC depletion causes stronger defects at later developmental stages, possibly indicating that previous results had been blurred by maternal contribution and protein perdurance. They further show that HAUS/Augmin depletion causes similar, but milder defects than that of gamma-TURC and speculate that it may be involved in a subset of gamma-TURC functions.

The mechanisms of microtubule nucleation and organization in neurites are of high importance to our understanding of neuronal cell biology but largely still unresolved. Whether gamma-TURC is a major factor has been debated, especially the Drosophila data have been inconclusive. This manuscript attempts a clarification by looking at another developmental period where analysis may be more conclusive. The solid, albeit somewhat superficial, analysis demonstrates an important role for gamma-TURC and its effector HAUS but also clearly shows other mechanisms must be at play as well. I have a few technical comments, and it would be nice to deepen the link between and HAUS/Augmin and microtubules a bit more.
Reviewer 1 Comments for the Author:
Major comments
1. The conclusion that gamma-TURC is a major nucleator in sensory neurons is based on the strong phenotype caused by a single RNAi construct against Grip91, all other RNAi lines cause milder defects. What is the evidence that this is not an off-target effect? The authors could address this by using another manipulation (e. g., in Ori-McKenney et al., 2012, the effect of

© 2024. Published by The Company of Biologists under the terms of the Creative Commons Attribution License (https://creativecommons.org/licenses/by/4.0/).

gamma-tub23C RNAi is enhanced by crossing in a gamma-tub23C mutant allele).

We agree that Grip91 RNAi produces a stronger effect than the other RNAi's, but the effect caused by these other RNAi's is not negligible. For example, expression of γ-tub23C-RNAi1, which was previously used in Ori-McKenney 2012, resulted in a ~47% reduction in arbor area and a ~39% reduction in dendrite tips. Indeed, we contend that 4/5 of the other RNAi lines also produce strong phenotypes in pupae relative to what had been observed previously in larvae. It is also true that the more severe phenotype caused by the Grip91-RNAi construct could be due to an off-target effect, but based on its sequence there are no predicted off- target genes (as stated on the VDRC website). Moreover, I'm not sure the reviewer's suggestion would reveal an off-target effect. Enhancement of the phenotype by including a mutant allele would occur whether or not there is an off-target effect, as RNAi depletion is likely incomplete (as suggested by our new RT-qPCR analysis (new Figure S2). We think the only possible way rule out an off-target effect would be to ectopically express an RNAi- resistant rescue construct containing many silent mutations (it is a long-hairpin RNAi construct). But interpreting the results would not be trivial, as over-expression of the rescue construct may generate phenotypes, as the endogenous γ-TuRC genes are expressed at low levels. Indeed, over-expression of γ-tubulin-GFP results in artefactual localisation patterns (Mukherjee et al., 2020, eLife). It is common practice by prominent members of the field to use more than one RNAi line (rather than rescue constructs) to conclude that the effects observed are caused by a depletion of the gene in question (e.g. when examining the role of Msps in dendrite pruning (Tang et al., 2023, EMBOJ), and when examining the role of Patronin in class I da neuron dendrites (Feng et al., 2019, JCB)). Thus, we believe that our use of multiple RNAi constructs is in line with other publications. Nevertheless, we now clearly point out in the Results section that we cannot rule out off-target effects contributing to the phenotype (lines 203-216):

*"As expected, phenotypic strength varied, presumably due to differences in RNAi efficiency, with the strongest reduction in both perimeter area and tip number resulting from expressing Grip91^{GCP3}-RNAi1 (corresponding to VDRC KK library construct 104667) and the second strongest resulting from expressing γ-tubulin23C-RNAi1, which was previously used when assessing phenotypes in larvae (Ori-McKenney et al., 2012) (Figure 1B-D). **Although we cannot rule out that the stronger phenotype observed with Grip91^{GCP3}-RNAi1 is due to unintended depletion of another gene**, Grip91^{GCP3}-RNAi1 has no predicted off-targets (VDRC website). Moreover, we confirmed by RT-qPCR on wing disc samples that Grip91^{GCP3}-RNAi1 was able to deplete Grip91 mRNA (Figure S2) and that expression in class I da neurons strongly reduced the presence of endogenously-tagged γ-tubulin-GFP at Golgi mini-stacks within the soma (Figure S3). Thus, expressing Grip91^{GCP3}-RNAi1 has a strong effect on γ-TuRCs in da neurons."*

2. The authors should try to tie the gamma-TURC phenotype at the pupal stage to microtubules, e.g., by measuring the microtubule density (using EB1GFP or similar).

We agree that this would have been a nice experiment to perform, but the first author capable of imaging EB1-GFP comets in pupae has long since left and we currently don't have the necessary expertise in the lab. As this would be a technically challenging protocol to develop from scratch, we decided to focus on other experiments for the revised manuscript. We hope that the Reviewer will agree that γ-TuRCs have never been implicated in processes other than those related to microtubules and that our analysis of multiple γ-TuRC RNAi lines would strongly indicate that γ-TuRCs are required for dendritic growth and branching, presumably via their role in regulating microtubules. We have, however, increased our analysis of EB1-GFP comets in larval neurons to make a strong link between Augmin depletion and defects in microtubule polarity and frequency.

3. To ascertain that HAUS/Augmin acts via its canonical gamma-TURC based mechanism, the authors should address the genetic relationship between the two complexes. Does co- depletion of gamma-TURC and HAUS leads to alterations of the dendrite defects that can be interpreted? Or does gamma-tub overexpression suppress Augmin LOF?

Results from these kinds of genetic interactions are very difficult to interpret. Given that depletion via RNAi is typically incomplete (also suggested by our new RT-qPCR data), an enhancement in phenotype from a double depletion would occur *both* if Augmin worked with γ-TuRCs or independently of γ-TuRCs, so we would not be able to distinguish between these possibilities. As for over-expressing γ-tubulin to suppress Augmin depletion, we already know that γ-tubulin-GFP overexpression results in artefactual localisation of γ-tubulin-GFP (Mukherjee et al., 2020) and because γ-TuRCs are multi-protein complexes, it is unlikely that over-expressing just

© 2024. Published by The Company of Biologists under the terms of the Creative Commons Attribution License (https://creativecommons.org/licenses/by/4.0/).

one component would produce more functional complexes. We believe the only way to test this properly would be to colocalise single particles of Augmin and γ-TuRC but this would be extremely challenging *in vivo* (as we discuss below) due to low signal-to- noise and the depth of imaging (which excludes TIRF imaging). Thus, in the absence of a feasible experiment to directly show that HAUS/Augmin acts via its canonical gamma-TURC based mechanism, we have toned down the text to accommodate this concern. We also point out that, to our knowledge, a γ-TuRC-independent role for Augmin has not been reported previously.

In the abstract…(lines 38-41)

"Thus, γ-TuRCs are essential for various aspects of complex dendritic arbor development, and **they appear** to function in higher-order branching via the Augmin pathway, which promotes the elaboration of dendritic arbors to help define neuronal morphology."

In the introduction….(lines 168-172):

*"Given the known role and mode of operation of Augmin in other cell types, our findings **suggest** that Augmin mediates sparsely distributed microtubule nucleation events throughout the dendritic arbor of class IV da neurons that provide sufficient numbers of microtubules to stabilise the nascent growth of higher-order dendrites."*

In the discussion….(lines 422-430):

*"**While we cannot exclude the possibility that Augmin functions independently of γ-TuRCs in da neurons, a γ-TuRC-independent function of Augmin has not been reported to our knowledge**. Given the effects we observe on microtubule polarity and frequency after Augmin depletion, our data are fully **consistent with** Augmin functioning to recruit γ-TuRCs to the sides of microtubules to mediate microtubule nucleation events, as occurs in cycling cells (Song et al., 2018). We propose that these nucleation events occur throughout the arbor and lead to the anterograde growth of microtubules within terminal dendrites, which in turn help to stabilise the nascent growth of these dendrites."*

4. Localization of augmin would be interesting to know. But not a must.

This would indeed be very interesting but also very challenging. Augmin functions as a single particle during a microtubule nucleation event and each Augmin component exists as a single molecule per complex. Thus, it would be necessary to detect a single protein above background/noise in a neuron in vivo. We certainly cannot detect individual γ-TuRCs in these neurons when using γ-tubulin-GFP, even though there are 14 molecules per γ-TuRC. Over-expressing a GFP-tagged Augmin component using the Gal4-UAS system may generate fluorescent puncta, but we would be sceptical that these represent genuine localisation patterns. Thus, we hope the reviewer will agree that mapping Augmin localisation in class IV neuron dendrites is beyond the scope of this study.

5. Do higher order dendrite fail to form in Augmin LOF? Or do they form and retract? Could be addressed in a timelapse experiment.

This is a very good question. We have now imaged larval class IV neurons live (control vs Augmin-RNAi) for 30 minutes and quantified the rate of new terminal dendrite formation (per 100¤m) and the percentage of terminal dendrite loss over 30 minutes. The results are displayed in **Figure 4I,J** and show that new dendrite formation is unaffected in Augmin-RNAi (as expected if Actin reorganisation is what promotes nascent branch assembly) but that **terminal dendrite retention is strongly affected**, with ~40% of unstable dendrites in Augmin- RNAi compared with 23% in controls (p=0.004). In combination with our new data on EB1- GFP comet reduction in terminal dendrites (Figure 4H), the results from this experiment suggest that growing microtubules within terminal dendrites generated in an Augmin- dependent manner help stabilise nascent dendrite growth (as we known microtubule growth in these terminal dendrites correlates with dendrite stability (Ori-McKenney et al., 2012). We feel this significantly strengthens the paper and we thank the Reviewer for suggesting the experiment.

6. Figs. 1E, F. Does the ratio - branchpoints per um - differ?

Good question. It's actually relevant to both the experiments in Figure 1 (Grip91-RNAi) and Figure 3 (Dgt4-RNAi and Grip71-RNAi), so we have done this for both. We had originally only measured the length of the longest primary dendrite and the number of major branchpoints leading to a significant portion of the arbor. These measurements were not appropriate to calculate the branchpoint per um ratio. We therefore measured the combined length of primary and secondary dendrites (that make up the main skeleton of the arbor) and the total number of primary and

© 2024. Published by The Company of Biologists under the terms of the Creative Commons Attribution License (https://creativecommons.org/licenses/by/4.0/).

secondary branchpoints. Using these data, we calculated primary/secondary dendrite lenght per branchpoint (**new Figures 1H, 3G**). This shows major branchpoints occur more frequently per major dendrite length in Grip91-RNAi neurons, suggesting that while the primary and secondary dendrites of Grip91-RNAi neurons struggle to both grow (new Figure 1F) and branch (new Figure 1G) they perhaps struggle more to grow than to branch. For Dgt4-RNAi and Grip71-RNAi neurons, there is a relatively small reduction in total primary and secondary dendrite length (new Figure 3E) but the number of primary and secondary branchpoints does not change. Thus, the primary and secondary dendrites also struggle more to grow than to branch, but they do not struggle nearly as much as Grip91- RNAi neurons. Note that we have retained the measurements for primary branch length (Figures 1E, 3D) but replaced the frequency distribution graphs of major branchpoints with those of primary and secondary branchpoints (Figures 1G, 3F).

Minor comments

1. Some references are incorrect. References for PNS neuron pruning: Kuo, Jan & Jan 2005, Williams & Truman 2005, but not Williams and Shepherd 1999. First reference describing dendrite regrowth: Kuo, Jan & Jan 2005

Thanks for pointing this out – now corrected.

2. Line 150 "Although da neurons are rarely analysed in pupae…" is a bit off. Though not cited here, there are a number of papers that look specifically at this stage to study dendrite regenerative and/or growth mechanisms

We apologise for this, you are correct. We have changed this to *"Although microtubule regulation in da neurons is normally analysed in larvae…"*

3. Figs 1F, 3E. Can the data for the different genotypes be displayed next to each other? Above each other, the data is harder to interpret. Also please add more percentage points on the y axis

Thanks for pointing this out, we have changed the graphs accordingly.

*****

Reviewer 2 Advance Summary and Potential Significance to Field:

In this manuscript Mukherjee and colleges address the role of Augmin and gamma- tubulin in Drosophila dendrite development. Although Augmin function in neurons has been described before (mainly) in cultured mammalian neurons, addressing its role in another system and importantly in vivo is relevant.

Reviewer 2 Comments for the Author:

Here the authors found that gamma-tubulin is more broadly required during development whereas Augmin is mainly required for the fine/distal branching. As Augmin is needed to nucleate microtubules from existing microtubules they looked at microtubule polarity and found some subtle defects, a bit surprisingly mainly in a more proximal branch.

In general the work is well performed. However I think that the authors should take it one step further to try to link the morphological defects to the microtubule defects. e.g. is microtubule density affected in the terminal branches, where does Augmin localize. Now the authors make the point that there are fewer terminal branches with EBs, however this is a very small effect and not significant, so not convincing this is a real effect of KD.

We appreciate the Reviewer's concern that is important to link Augmin depletion to defects in microtubules. For this, we have increased our analysis of EB1-GFP comets in the different areas of the dendritic arbor and rearranged the graph in **Figure 4H** to better visualise the effect in terminal dendrites. The data now show a clear reduction in the frequency of EB1- GFP comets in terminal dendrites, while there is no reduction in secondary and tertiary dendrites (now grouped due to their similarity concerning EB1-GFP comets). We have now removed the graph measuring the proportion of terminal dendrites containing at least one EB1-GFP comet, as we realised this did not provide a true representation of the effect of Augmin depletion (because a dendrite with 2, 3, or 4 comets is scored the same as a dendrite with just 1 comet). In summary, our data now show a clear and significant reduction in the frequency of EB1-GFP comets in terminal dendrites (**Figure 4H**), which fits with our new data that terminal dendrites are unstable (new Figure 4J), and with our original data that there are fewer dendritic tips after Augmin depletion (Figure 3C,

© 2024. Published by The Company of Biologists under the terms of the Creative Commons Attribution License (https://creativecommons.org/licenses/by/4.0/).

Figure 4B,D).

One other point that needs revision are the graphs in 1F and 3E. This is not the correct representation, e.g. in 3E I don't know if the Dgt4 in bin 2 should be read as 80% or as 30%. Either these need to be separated (like e.g. 4F) or the data should differently represented such that 1 condition goes to 100% (so column 1 control, column 2 KD1, …). Also the statistics are missing for this data.

We agree and this was also pointed out by Reviewer 1 – we have now changed the graphs to separate the bars. We have now performed a two-sample Kolmogorov-Smirnov test for Figure 1F (significant difference) and a Kruskal-Wallis test for Figure 3E (no significant difference) and added the p values above the graphs.

Textual

I appreciate the efforts of the authors to make the text accessible to non- drosophila readers. However this could still be improved a bit, e.g. the testing of the KK library can maybe be placed in the methods and along the text I forgot that Grip71 = NEDD1

As suggested, we have moved the part about testing the KK lines for the 40D insertion to the methods. We have also replaced "Grip71" with "Grip71^{NEDD1}" throughout the text.

line 320 "these data … secondary dendrites" I don't follow how this is inferred from the results describe above which are quantifications of the terminal branching.

Sorry for the confusion. The results above also quantify the peripheral area, which is a measure of how well the primary and secondary dendrites grow. As the peripheral area is not affected at 120h AEL in Augmin-RNAi neurons, while tip number is reduced by ~22%, we infer that Augmin depletion perturbs terminal branches more than primary and secondary branches.

Line 343 It would make it easier for the reader to only describe the MT polarity as xx % anterograde (or retrograde) in the paragraph

Thanks for this suggestion. We have now reworded the paragraph to make it much simpler and also grouped distal secondary and tertiary dendrites into one category (our new data showed that polarity was affected similarly in secondary and tertiary dendrites). This part of the paragraph now reads (lines 356-366):

"When filming the more distal regions of control arbors, however, we could identify differences in comet polarity and frequency. In secondary and tertiary dendrites (see Figure S1B for how dendrite order was established), there was a significant reduction in the proportion of retrograde comets in Augmin-RNAi neurons: 73.8% (220/298) and 60.9% (380/622) of comets were retrograde in control-RNAi or Augmin-RNAi neurons, respectively (p<0.001) (Figure 4G). Thus, Augmin depletion can affect microtubule polarity in Drosophila dendrites, as it does in mammalian axons (Cunha-Ferreira et al., 2018; Sánchez-Huertas et al., 2016). In contrast to secondary and tertiary dendrites, however, a higher frequency of retrograde comets was observed in the terminal dendrites of Augmin-RNAi neurons compared to control neurons, although this was not statistically significant (Figure 4G)"

Line 392 what is the "wac mutant"?

Apologies for not being clear. Wac is a member of the Augmin complex. The sentence now reads (lines 404-407):

"This is consistent with a previous observation that embryonic class I ddaE neurons within augmin mutant flies also show no difference in dendrite tip number compared to controls (Yalgin et al., 2015)"

Line 393-398 : "In control … Augmin depletion" I think the point does not come across well. And lost the detailed comparison

We have modified the text to read (lines 407-411):

"In control-RNAi and Augmin-RNAi larval class I neurons, we measured an average of ~36 dendrite tips at 120h AEL, while Yalgin et al. measured an average of ~20 dendrite tips during stage 17 of embryogenesis (just before hatching into larvae), ~96 hours earlier. Thus, class I ddaE neurons add, on average, ~16 new dendrite branches during ~96 hours of larval development and this does not require Augmin"

*****

Reviewer 3 Advance Summary and Potential Significance to Field:

© 2024. Published by The Company of Biologists under the terms of the Creative Commons Attribution License (https://creativecommons.org/licenses/by/4.0/).

Neurons have elaborate dendritic arbors. These are arbors are microtubule-rich, raising the question of how the microtubule cytoskeleton might influence dendrite morphogenesis. In this manuscript, the authors focus on the role that the nucleation of new microtubules by augmin and gamma-TuRC components might play in dendrite branch formation and, ultimately, shaping arbor structure. While both augmin and gamma-TuRC have been studied for many years, there has been the outstanding question of whether the fairly mild (or lack of) neuronal phenotypes may reflect an incomplete reduction of their activity. A strength of this current study is the use of a dendrite re-growth assay during late stages of development to uncover any latent defects in dendrite morphogenesis. This manuscript nicely presents the dendrite morphogenesis phenotypes that result from knocking-down components of the augmin and gamma-TuRC complexes (it is a nice descriptive study). Overall, this is a very nice body of work, with just a few major concerns.

Reviewer 3 Comments for the Author:

1. The authors describe the use of the dendrite re-growth assay during pupal stages as an advantageous model to maximize the effects of RNAi-mediated knockdown (and depletion of any maternal contribution). But it is not clear whether the RNAi strains effectively knockdown protein levels. Can the authors assay the efficacy of the different RNAi strains to deplete the target proteins? For example, perhaps it is possible to use a broadly expressed Gal4 (such as elav-Gal4, or tub-Gal4) to express the RNAi constructs and then use western blots to assay the degree of knockdown. Doing this analysis at larval and pupal stages would potentially bolster the author's model that dendrite re-growth is affected because protein levels are more reduced during pupal v. larval stages.

We agree with the reviewer that knowing the efficiency of RNAi at the protein level at different stages would have been very informative. Unfortunately, however, we do not have antibodies for either Grip91 or Dtg4. It may also be difficult to interpret data from all neurons in terms of whether maternally supplied proteins are stable specifically in da neurons. Nevertheless, in an attempt to address the concern to the best of our ability, **we used to RT- qPCR on wing-disc samples to test whether Grip91 and Dtg4 mRNA levels could be depleted using their respective RNAi constructs**. We chose wing discs as a member of our new team had experience performing RT-qPCR with wing discs and we reasoned that both Augmin and Grip91 mRNA should readily be transcribed in this dividing tissue. The data can be found in **Figure S2**. We performed 3 biological replicates (mRNA samples from 3 different lots of 50 wing discs) and repeated the reverse transcription 3 times on each sample. This gave us 9 data points each for control, Grip91-RNAi and Dgt4-RNAi. From this, we calculated a ~42% and ~31% reduction for grip91 and dgt4 mRNA, respectively. Thus, we can conclude that both RNAi constructs can target and deplete their respective gene. Of course, these depletions are not complete and this may influence the strength of the phenotypes we observe in da neurons, although it is important to note that these measurements were made using a different Gal4 driver in a different cell type and so may not accurately reflect the level of depletion in da neurons. They just tell us that the RNAi constructs are capable of depleting their target genes. Importantly, however, expressing Grip91-RNAi in class I da neurons leads to the loss of γ-tubulin-GFP signal at the Golgi stacks in the soma (Figure S3), which strongly perturbs microtubule nucleation (data for another story), so expressing Grip91-RNAi in da neurons clearly has a major effect on γ-TuRCs, despite there being “only” a ~42% reduction in mRNA in wing discs. Moreover, the RNAi lines we use for the majority of the paper produced the strongest phenotypes in pupal da neurons despite having no predicted off- target genes. We hope the Reviewer will therefore agree that we can be confident that the RNAi lines are effective, but that it is not currently feasible to compare protein levels after RNAi in da neurons at larval and pupal stages.

2. What is known about the expression and distribution of augmin and gamma-TuRC components in the class IV and class I da neurons? It would be helpful to include information about the expression (and potentially localization) of these components. For example, are augmin components not well-expressed in class I neurons? Do augmin complex members localize to dendrites?

In our Mukherjee et al., 2020 eLife paper, we carefully documented the expression and localisation of endogenously-tagged γ-tubulin23C-GFP in class I and class IV da neurons. We found that γ-tubulin23C-GFP localised to the multiple mini-Golgi stacks within the soma of both neuron types. In class I da neurons, γ-tubulin23C-GFP localised as puncta in some dendritic branchpoints

© 2024. Published by The Company of Biologists under the terms of the Creative Commons Attribution License (https://creativecommons.org/licenses/by/4.0/).

and within local expansions of the dendrite cell membrane that we called “dendritic bubbles”. In class IV neurons, apart from localising to a few Golgi outposts close to the soma, the γ-tubulin23C-GFP was diffuse throughout the arbor. We believe that this is consistent with individual γ-TuRCs being recruited to the sides of pre-existing microtubules via individual Augmin complexes, as it would be impossible to detect single γ- TuRCs above background using γ-tubulin23C-GFP (14 GFP molecules). We don’t have an endogenously-tagged fluorescent Augmin line, but even if we did it would also be impossible to detect single Augmin complexes within the dendrites as each Augmin component within the complex has a stoichiometry of 1. We believe that using UAS-controlled fluorescent reporters of γ-TuRCs or Augmin would result in artefacts caused by over-expressing a single component of a multi-protein complex that is normally expressed at low levels (as appears to be the case for UAS-γ-tubulin-GFP – Mukherjee et al., 2020).

Nevertheless, we were stimulated by the Reviewer’s comment to test whether Augmin may be expressed to higher levels in class IV neurons. For this, we crossed a Dgt5 Gal4-trap line (where Gal4 has been inserted into the Dgt5 gene region) to UAS-Myr-GFP (fluorescent membrane marker) and examined the GFP signal in dorsal clusters that include the da neurons. The data is shown in **Figure S4** and shows that Dgt5 is indeed expressed to higher levels in class IV da neurons compared to class I neurons. We believe this makes the paper stronger and we thank the Reviewer for their suggestion.

3. Is it possible to visualize gamma-Tub23C-GFP in dendrites and determine if its localization is disrupted by the knockdown of Grip91? This would support the authors' model that augmin is recruiting gamma-Tub/gamma-TuRC to positions of nascent branches in the arbor.

This would have been a nice experiment, but, as discussed in point 2 above, we cannot observe any specific γ-tubulin23C-GFP signal within the dendrites of class IV da neurons, except for the signal at a few proximal Golgi outposts, which will be unrelated to Augmin. Augmin and γ-TuRC normally act as individual complexes during Augmin-mediated microtubule nucleation (rather than accumulating at an MTOC) making it very difficult, if not impossible, to detect them as individual units in cells *in vivo*.

4. On a related note: The authors claim the "Augmin microtubule nucleation pathway as being the γ-TuRC-dependent..." (line 164), but no support for this is included in the manuscript. Is it possible to enhance the augmin phenotypes by reducing gamma- TuRC activity?

It was also pointed out by Reviewer 1 point 3 that we had not shown that Augmin functions via γ-TuRCs and they also suggested epistasis experiments. However, as discussed in the response to Reviewer 1, performing epistasis experiments with RNAi depletion that is not 100% efficient will not allow us to distinguish between whether Augmin functions with γ- TuRCs or not (an enhancement of the phenotype would occur in both cases). To our knowledge, Augmin has not been shown to function in any other way, but we have nevertheless toned down the text to accommodate this concern.

In the abstract…(lines 38-41)

“Thus, γ-TuRCs are essential for various aspects of complex dendritic arbor development, and **they appear** to function in higher-order branching via the Augmin pathway, which promotes the elaboration of dendritic arbors to help define neuronal morphology.”

In the introduction…(lines 168-172):

*“Given the known role and mode of operation of Augmin in other cell types, our findings **suggest** that Augmin mediates sparsely distributed microtubule nucleation events throughout the dendritic arbor of class IV da neurons that provide sufficient numbers of microtubules to stabilise the nascent growth of higher-order dendrites.”*

In the discussion….(lines 422-430):

*“**While we cannot exclude the possibility that Augmin functions independently of γ-TuRCs in da neurons, a γ-TuRC-independent function of Augmin has not been reported to our knowledge**. Given the effects we observe on microtubule polarity and frequency after Augmin depletion, our data are fully **consistent with** Augmin functioning to recruit γ-TuRCs to the sides of microtubules to mediate microtubule nucleation events, as occurs in cycling cells (Song et al., 2018). We propose that these nucleation events occur throughout the arbor and lead to the anterograde growth of microtubules within terminal dendrites, which in turn help to stabilise the nascent growth of these dendrites.”*

© 2024. Published by The Company of Biologists under the terms of the Creative Commons Attribution License (https://creativecommons.org/licenses/by/4.0/).

Minor Comments

5. For clarity, consider referring to Grip91-RNAi1 and Dgt4-RNAi as such throughout rather than switching to referring to them as "γ-TuRC-RNAi" and "Augmin-RNAi," respectively.

We appreciate the suggestion but prefer to stick to using "γ-TuRC-RNAi" and "Augmin-RNAi" so that non-specialists will better follow the paper.

6. Please include some representative images for the data represented in Figure 4H, in particular the distal terminal dendrites.

We have included representative movies (Movie S1,S2) to show the difference in EB1-GFP comets in terminal branches between control-RNAi and Augmin-RNAi neurons. We hope the Reviewer agrees that these movies are better representations than still images.

7. Line 227: Change "the authors" to "others."

Changed.

8. Line 445: Typo, change "forth" to "fourth."

Changed.

9. Line 446: Some part of the sentence must have been inadvertently deleted ("possibility is does not depend on microtubule growth…"); please remedy.

Thanks, we removed "is" to correct the sentence.

10. Line 489-490: Yang and Wildonger (2020) reference should be removed here; don't believe that it shows microtubule growth originating at Golgi outposts (?).

Due to a rearrangement of the Discussion, this text has now been lost from the manuscript anyway.

11. Figure 6D: The figure legend should clarify that this model represents "distal secondary branches" in the dendritic arbor; microtubule polarity was not affected in other parts of the arbor.

This is a good point, although we now also find polarity changes in distal tertiary branches after analysing more neurons. We have therefore included the following statement at the start of the figure legend (lines 829-831):

"Cartoons representing how Augmin-mediated microtubule nucleation may promote the formation of terminal dendrites from either secondary or tertiary dendrites in the distal region of the class IV da neuron."

Second decision letter

MS ID#: JOCES/2023/261534

MS TITLE: γ-TuRCs and Augmin are required for the development of highly branched dendritic arbors in Drosophila

AUTHORS: Amrita Mukherjee, Yaiza Andres Jeske, Isabelle Becam, Anaelle Taieb, Paul Brooks, joanna aouad, clementine monguillon, and Paul J Conduit

We have now reached a decision on the above manuscript.

To see the reviewers' reports and a copy of this decision letter, please go to: https://submit-jcs.biologists.org and click on the 'Manuscripts with Decisions' queue in the Author Area. (Corresponding author only has access to reviews.)

As you will see, the reviewers gave favourable reports but raised minor critical points that will require amendments to your manuscript. I hope that you will be able to carry these out because I would like to be able to accept your paper once it is returned.

© 2024. Published by The Company of Biologists under the terms of the Creative Commons Attribution License (https://creativecommons.org/licenses/by/4.0/).

Please ensure that you clearly highlight all changes made in the revised manuscript. Please avoid using 'Tracked changes' in Word files as these are lost in PDF conversion.

I should be grateful if you would also provide a point-by-point response detailing how you have dealt with the points raised by the reviewers in the 'Response to Reviewers' box. Please attend to all of the reviewers' comments. If you do not agree with any of their criticisms or suggestions please explain clearly why this is so.

Reviewer 1

Advance summary and potential significance to field

The gamma-tubulin ring complex (gamma TURC) is a component of many microtubule organizing centers and hence believed to be important. Somewhat surprisingly, gamma TURC manipulations in larval fly neurons yielded only minor defects in dendrite growth, even though this process must be microtubule-dependent. One explanation is perdurance of maternal material. The authors have here revisited this question and knocked down gamma-TURC and a related complex augmin in pupal fly neurons and show that the effects are more severe there. While their results still leave open the possibility of other gamma-TURC-independent mechanisms, they do show that gamma-TURC is important in some areas of dendritic arbors.

Comments for the author

A major question in this work was how important gamma-TURC is for dendrite development. Of the six RNAi lines used, all reduce the pupal dendritic tree somewhat, but only one strongly. This is in part blurred by the analysis, because the authors only measured the dendritic area but not dendrite length. So is Grip91 RNAi just the only effective one (possible), does it cause side effects, or does Grip91 have another role in dendrite development independent of gamma-TURC? The added controls clearly show Grip91 RNAi does reduce gamma-TURC, so that is good. But I would still ask to clearly state also in Fig 2 that the used RNAi was that against Grip91. The use of "gamma-TURC RNAi" is too bold. Similarly, please change "Augmin RNAi" in Fig. 4 to "Dgt4 RNAi". A EB1-GFP analysis would have been good because gamma-tubulin depletion in larval da neurons has mostly been reported to cause orientation defects, as opposed to defects in microtubule density. I would ask to acknowledge this in the discussion. The observation that Dgt4 RNAi causes a shift in microtubule density towards lower order dendrites, and not a general decrease, would be more consistent with a role in microtubule localization than in nucleation. There are also still a number of missing or wrong references. in the introduction, please add references for class 4 and class 1 neuron function (lines 114, 118). Williams & Shepherd, 1999 is not a reference for dendrite pruning, the correct reference is Williams and Truman, 2005. On the other hand, Williams and Truman, 2005 does not show dendrite regrowth.

Reviewer 2

Advance summary and potential significance to field

I appreciate the life imaging of terminal branches the authors did. These results seam to show that these branches are less stabilized upon Augmin depletion. This combined with the EB imaging suggest that Augmin is needed to maintain sufficient (plus end out) MTs to stabilize new terminals.

Comments for the author

I’m happy with the authors improvements and only have a few very minor points:

- 1G not completely clear what was done. Is this a representation of the sum of primary+secondary branch points? Main text suggests that both primary as well as secondary branch points are reduced. “There was also a strong reduction in the number of primary and secondary branchpoints”

© 2024. Published by The Company of Biologists under the terms of the Creative Commons Attribution License (https://creativecommons.org/licenses/by/4.0/).

- In Figure 3F: where does p=0.46 belong to? (there are 2 KD conditions)

- Error bars – Are those SD? And check consistency (line thickness varies in many cases e.g. 3D).

Reviewer 3

Advance summary and potential significance to field

The authors' study addresses the outstanding question of the role that augmin and γ-TuRCs play in neuronal development. The authors' data suggests that these molecular complexes are involved in the elaboration of neuronal dendrites.

Comments for the author

The authors have addressed the majority of the concerns, and the manuscript is quite nicely improved due to the authors' efforts. The new data, including data in the supplement, are great additions.
One minor comment: Consider changing the red-green color combination for Figure 4J to be color-blind friendly.

Second revision

Author response to reviewers' comments

Response to Reviewers:

Reviewer 1:

- I would still ask to clearly state also in Fig 2 that the used RNAi was that against Grip91. The use of "gamma-TURC RNAi" is too bold. Similarly, please change "Augmin RNAi" in Fig. 4 to "Dgt4 RNAi".
  We have now made these changes in the figures and throughout the text.
  A EB1-GFP analysis would have been good because gamma-tubulin depletion in larval da neurons has mostly been reported to cause orientation defects, as opposed to defects in microtubule density. I would ask to acknowledge this in the discussion. We have included the following statement in the Discussion: "*Thus, future studies may consider analysing phenotypes in pupae as well as embryos or larvae. For example, examining EB1-GFP comets in pupal neurons depleted for γ-TuRCs would be informative, as previous studies in larvae report relatively minor changes, or no changes, in EB1-GFP comet frequency after γ-tubulin depletion (Nguyen et al., 2014; Ori-McKenney et al., 2012). Moreover, one may see stronger effects on microtubule dynamics after Augmin depletion.*"
- The observation that Dgt4 RNAi causes a shift in microtubule density towards lower order dendrites, and not a general decrease, would be more consistent with a role in microtubule localization than in nucleation.
  This is an interesting take on the data. We have now modified the discussion of this result as follows: "*The increase in comet frequency in distal secondary and tertiary dendrites could be due to the induction of neuronal stress, as is the case after the depletion of other microtubule regulators (Feng et al., 2019). Alternatively, the reduction in microtubule growth in terminal dendrites may increase the availability of tubulin dimers for microtubule growth in distal secondary and tertiary dendrites. In either case, we conclude that Augmin is required to maintain proper microtubule polarity in distal secondary and tertiary dendrites and to promote anterograde microtubule growth within terminal dendrites.*"

© 2024. Published by The Company of Biologists under the terms of the Creative Commons Attribution License (https://creativecommons.org/licenses/by/4.0/).

- There are also still a number of missing or wrong references. in the introduction, please add references for class 4 and class 1 neuron function (lines 114, 118).
  We apologise for these errors. We have now added the following references: for class I neurons: (Song et al., 2007; Vaadia et al., 2019); for class IV neurons: (Hu et al., 2017; Xiang et al., 2010; Zhong et al., 2010) for their nociceptive function, and (Grueber et al., 2002) when stating they tile the body wall by branching extensively. Note that we did not add all possible references that relate to class IV neurons being nociceptive as there are many. We therefore added "e.g." before the references to make it clear that these are not the only papers showing that class IV neurons are nociceptive.
  Williams & Shepherd, 1999 is not a reference for dendrite pruning, the correct reference is Williams and Truman, 2005. On the other hand, Williams and Truman, 2005 does not show dendrite regrowth.
  We have now changed the references accordingly.

Reviewer 2:
I'm happy with the authors improvements and only have a few very minor points:
- -1G not completely clear what was done. Is this a representation of the sum of primary+secondary branch points? Main text suggests that both primary as well as secondary branch points are reduced. "There was also a strong reduction in the number of primary and secondary branchpoints"
  We apologise for the confusion. Yes, it is the sum of primary and secondary branch points. We have now re-worded the text to make this clearer: "*There was also a strong reduction in the <u>combined total</u> number of primary and secondary branchpoints (Figure 1G)*"
- -In Figure 3F: where does p=0.46 belong to? (there are 2 KD conditions)
  This p value was generated by an ANOVA analysis that tested whether the medians varied significantly (when considering all 3 datasets). We acknowledge that this was not clear so we have now added the p values for each dataset from a "multiple comparisons" analysis performed within Graphpad Prism. We also changed the legend text to: "*A Kruskal-Wallis test was used to compare each condition to the control.*" This also made us realise that there was a mistake in the legend as we had included the statement: "*A two-sample Kolmogorov-Smirnov test was used to compare the datasets*", which has now been removed.
- -Error bars – Are those SD?
  Most errors bars correspond to 95% confidence intervals (CIs) and this information is indicated in the figure legends
  And check consistency (line thickness varies in many cases e.g. 3D)
  Thanks for pointing this out, we have now homogenized the line thicknesses in all figures.

Reviewer 3:
- Consider changing the red-green color combination for Figure 4J to be color-blind friendly.
  Thanks for pointing this out. We have now changed the colours.

<u>Third decision letter</u>

MS ID#: JOCES/2023/261534

MS TITLE: γ-TuRCs and Augmin are required for the development of highly branched dendritic arbors in Drosophila

AUTHORS: Amrita Mukherjee, Yaiza Andres Jeske, Isabelle Becam, Anaelle Taieb, Paul Brooks, joanna aouad, clementine monguillon, and Paul J Conduit
ARTICLE TYPE: Research Article

Thank you for the quick turn around. I am happy to tell you that your manuscript has been accepted for publication in Journal of Cell Science, pending standard ethics checks.

© 2024. Published by The Company of Biologists under the terms of the Creative Commons Attribution License (https://creativecommons.org/licenses/by/4.0/).
